# Inhibition of the Intrinsic but Not the Extrinsic Apoptosis Pathway Accelerates and Drives Myc-Driven Tumorigenesis Towards Acute Myeloid Leukemia

**DOI:** 10.1371/journal.pone.0031366

**Published:** 2012-02-29

**Authors:** Kari Högstrand, Eduar Hejll, Birgitta Sander, Björn Rozell, Lars-Gunnar Larsson, Alf Grandien

**Affiliations:** 1 Center for Infectious Medicine, Department of Medicine, Karolinska Institutet, Karolinska University Hospital, Stockholm, Sweden; 2 Department of Microbiology, Tumor and Cell Biology (MTC), Karolinska Institutet, Stockholm, Sweden; 3 Divisions of Clinical Research Center and Pathology, Department of Laboratory Medicine, Karolinska Institutet, Karolinska University Hospital, Stockholm, Sweden; 4 Center for Hematology and Regenerative Medicine, Department of Medicine, Karolinska Institutet, Karolinska University Hospital, Stockholm, Sweden; Emory University, United States of America

## Abstract

Myc plays an important role in tumor development, including acute myeloid leukemia (AML). However, MYC is also a powerful inducer of apoptosis, which is one of the major failsafe programs to prevent cancer development. To clarify the relative importance of the extrinsic (death receptor-mediated) versus the intrinsic (mitochondrial) pathway of apoptosis in MYC-driven AML, we coexpressed MYC together with anti-apoptotic proteins of relevance for AML; BCL-X_L_/BCL-2 (inhibiting the intrinsic pathway) or FLIP_L_ (inhibiting the extrinsic pathway), in hematopoietic stems cells (HSCs). Transplantation of HSCs expressing MYC into syngeneic recipient mice resulted in development of AML and T-cell lymphomas within 7–9 weeks as expected. Importantly, coexpression of MYC together with BCL-X_L_/BCL-2 resulted in strongly accelerated kinetics and favored tumor development towards aggressive AML. In contrast, coexpression of MYC and FLIP_L_ did neither accelerate tumorigenesis nor change the ratio of AML versus T-cell lymphoma. However, a change in distribution of immature CD4^+^CD8^+^ versus mature CD4^+^ T-cell lymphoma was observed in MYC/FLIP_L_ mice, possibly as a result of increased survival of the CD4+ population, but this did not significantly affect the outcome of the disease. In conclusion, our findings provide direct evidence that BCL-X_L_ and BCL-2 but not FLIP_L_ acts in synergy with MYC to drive AML development.

## Introduction

Acute myeloid leukemia (AML) represents a clonal expansion of hematopoietic stem and myeloid progenitor cells that have undergone malignant transformation [1], [2]. The leukemic cells are typically blocked in their differentiation and exhibit an abnormal extension of life span. Clinically it is a heterogeneous group of tumors. However, genetic studies during recent years have highlighted two common types of genetic changes, named class I and II, in AML [1], [2]. Class I comprise point mutations in genes involved in signal transduction, such as Ras, FLT3 and Kit, which control cell growth, survival and migration. Class II include a number of different translocations that all involves transcription factors such as MLL, AML1, CBF, EVI1 and RARα, which control the myeloid differentiation program, resulting in fusion proteins with aberrant activities [1], [2]. A common target gene for the class II translocation products and for deregulated signal transduction caused by some class I mutations is the oncogene/transcription factor MYC [3], [4]. MYC controls numerous genes involved in stem cell functions, differentiation, growth, immortalization, metabolism, survival and other fundamental cellular processes, and deregulated expression of MYC is very often implicated in human tumor development including hematological malignancies [5], [6]. For instance in Burkitt's lymphoma, MYC expression is deregulated through chromosomal translocations juxtaposing MYC and one of the immunoglobulin loci. Amplifications of *MYC* family genes (*MYC*, *MYCN*, *MYCL*) are common in a number of tumor types and are also found in AML although at a low frequency [5], [6]. Trisomy of chromosome 8 (where *MYC* resides) is common in AML, and the resulting increased *MYC* gene dosage has been suggested as a leukemogenesis mechanism [7]. In addition, both *MYC* and *MYCN* are frequently overexpressed in AML as a result of oncogenic events such as the class I and II mutations mentioned above [8], [9], [10]. As a consequence, the tumorigenic effect of the MLL-ENL translocation, one of the most devastating translocations in AML, was shown to be dependent on MYC expression [4]. Further evidence linking MYC to AML are the findings that enforced expression of MYC or MYCN in hematopoietic stem cells induce AML in mouse models [10], [11].

Overexpression of MYC in cells in culture or in animal models fuels cell growth and proliferation but is also a potent trigger of the apoptotic machinery, which acts as a failsafe mechanism to suppress tumorigenesis [5], [6]. Both the intrinsic (mitochondrial) pathway and the extrinsic (death receptor-mediated) pathways of apoptosis have been shown to be affected by deregulated MYC expression [12], [13]. Firstly, Myc is known to induce p19Arf, which activates p53, one of the main pathways controlling apoptosis [14]. p53 frequently becomes mutated during progression of MYC-induced tumors and targeted deletion of p53 accelerates MYC-induced B-lymphoma development in the Eμ-*myc* transgenic mouse model [15]. Secondly, MYC suppresses the expression of the anti-apoptotic proteins such as BCL-X_L_ and BCL-2, which regulate the intrinsic pathway. This mechanism is often bypassed during lymphoma progression of Eμ-*myc* mice [16]. Further, overexpression of BCL-2 or BCL-X_L_ in these mice [17], [18] or in transplanted bone marrow cells [11], [19] accelerates MYC-driven tumor development, suggesting that BCL-2 and BCL-X_L_ act in synergy with MYC in tumorigenesis. Moreover, Myc induces expression of pro-apoptotic proteins such as Bim and Bax [20], [21]. Thirdly, MYC sensitizes cells to death receptor-mediated apoptosis mediated by Fas-ligand, TNF-α or TRAIL [12], [13], [22], [23]. One reason for this could be that MYC upregulates expression of the TRAIL receptor DR5 and the Fas-ligand [5], [23], [24]. Further, epigenetic silencing of the caspase 8, which is the downstream effector of the death receptors, has been implicated in escape from apoptosis in *MYCN*-amplified neuroblastoma [25]. In support of this view, cells lacking Fas or TRAIL-R show increased survival in the Eμ-*myc* lymphoma model [26], [27]. Part of the increased response to death receptor signaling could be accounted for through MYC-induced suppression of NFκB and activation of Bak, facilitating caspase 8-mediated activation of Bax [28]. FLIP (CFLAR), another important regulator of death receptor-mediated apoptosis [29], inhibits apoptosis induced by death receptors of the TNFR superfamily by binding to caspase 8 at the death-inducing signaling complex (DISC) thereby blocking its activation [30]. FLIP can act as a tumor progression factor by promoting tumor establishment and growth *in vivo* [31], [32], [33]. Overexpression of MYC in myeloid cell lines has been shown to cause premature down regulation of FLIP_L_, thereby triggering the death receptor pathway [34]. Overexpression of FLIP_L_ in these cells could completely rescue the cells from apoptosis [34]. Further, MYC was shown to repress FLIP_L_ expression in a number if cells types both in culture and in vivo by binding to the *FLIP_L_* gene promoter region, resulting in increased sensitivity to TRAIL-induced apoptosis [35]. Several of these pathways are important for AML development. The class I mutations described above synergize with class II translocations at least in part by suppressing apoptosis, for instance by activating the RAS, MAPK and PI3 kinase pathways [1]. Overexpression of BCL2, BCL-X_L_ and/or MCL-1 and reduced expression of Bax is frequently observed in AML. AML cells are often resistant to TRAIL- and Fas-induced apoptosis, which has been attributed to death receptor or FADD downregulation, caspase 8 mutations or overexpression of FLIP_L_ [36].

Although the contribution of the extrinsic pathway to MYC-induced apoptosis has been studied extensively in different cell types in culture, only a few studies of the importance of this pathway for MYC-driven tumorigenesis in vivo have been performed (26, 27, 50), all using B- or T-lymphoma models. The potential impact of this pathway on MYC-induced AML is therefore unknown. In particular, the relative importance of the intrinsic and the extrinsic anti-apoptotic pathways in tumorigenesis fueled by MYC in vivo, including AML, remains to be determined.

The aim of this work was to assess the *in vivo*-impact of these two apoptotic pathways during MYC-induced malignant transformation of hematopoietic stem cells. We therefore coexpressed MYC together with anti-apoptotic gene products of either the extrinsic death receptor-mediated pathway (FLIP_L_) or the intrinsic (mitochondrial) pathway of apoptosis (BCL-2 or BCL-X_L_) in hematopoietic stem cells (HSC) followed by transplantation of the cells into syngeneic recipient mice. Expression of MYC alone in HSC resulted in development of both myeloid and T-lymphoid tumors within two months after transplantation. Expression of MYC together with BCL-X_L_ or BCL-2 resulted in almost immediate development of AML-like disease. Surprisingly and contrary to our expectations, expression of MYC together with FLIP_L_ did not accelerate tumorigenesis. However, while the ratio of AML versus T-cell lymphoma was not altered, the incidence of mature CD4^+^ T-cell lymphoma increased at the expense of immature CD4^+^/CD8^+^ T-cell lymphoma in MYC/FLIP_L_ mice. In conclusion, anti-apoptotic proteins of the intrinsic pathway accelerate the development of MYC-driven hematopoietic tumors to a larger extent than those of the extrinsic pathway of apoptosis.

## Materials and Methods

### Cell lines and mice

The human retroviral packaging cell line Phoenix-Eco (kindly provided by Dr. G. P. Nolan, Stanford University, CA, USA) was grown as described [37]. Female age-matched (6–8 weeks) inbred DBA/2 and BALB/c mice were purchased from Taconic (Lille Skensved, Denmark) or from the animal facility at the Department of Microbiology, Tumor and Cell Biology, Karolinska Institutet. This study was carried out in strict accordance with the recommendations in the Guide for the Care and Use of Laboratory Animals of the Swedish Board of Agriculture and the Department of Microbiology, Tumor and Cell Biology (MTC), Karolinska Institutet. The protocol was approved by the Ethical Committee of the Swedish board of Agriculture, North of Stockholm (Permit Number N99/11). All efforts were made in order to minimize animal suffering.

### Expression vectors

The pMSCV-IRES-EYFP vector was produced by replacing the enhanced green fluorescent protein (EGFP) of the pMSCV-IRES-EGFP vector (kindly provided by Dr. A. Nienhuis, St. Jude Children's Research Hospital, Memphis, USA) with the enhanced yellow fluorescent protein (EYFP) of pEYFP (Clontech, Palo Alto, CA) using the *NcoI* and *NotI* sites. The pMSCV-BCL-X_L_-IRES-EGFP vector was obtained by subcloning the *EcoRI* fragment containing human BCL-X_L_ from the pLXIN-BCL-X_L_ expression vector [38] into pMSCV-IRES-EGFP. The pMSCV-FLIP_L_-IRES-EGFP vector was generated as described previously [39]. MYC was isolated by PCR from cDNA from muscle cells from the Human Multiple Tissue cDNA (MTC) Panel 1 (Clontech) using the primers 5′-acgtgaattccaccatgcccctcaacgttagcttc and 5′-tacgtctcgagcttacgcacaagagttccgtag and subsequently cloned into the *EcoRI* and *XhoI* sites of pMSCV-IRES-EYFP to obtain the pMSCV-MYC-IRES-EYFP expression vector. pMSCV-BCL-2-IRES-EGFP was obtained by subcloning human BCL-2 from the cDNA clone MGC:21366 into the EcoRI site of pMSCV-IRES-EGFP.

### Production of retroviral particles

Ten micrograms of the plasmids pMSCV-IRES-EGFP (GFP), pMSCV-IRES-EYFP (YFP), pMSCV-BCL-X_L_-IRES-EGFP (BCL-X_L_-GFP), pMSCV-BCL-2-IRES-EGFP (BCL-2-GFP), pMSCV-FLIP_L_-IRES-EGFP (FLIP_L_-GFP) or pMSCV-MYC-IRES-EYFP (MYC-YFP) expression vectors were used to transiently transfect Phoenix-Eco packaging cell line using Lipofectamine 2000 Reagent (Invitrogen Ltd, Paisley UK). Supernatants containing recombinant viral particles were harvested 48 and 72 hours after transfection, passed through a 0.45 µ M filter, and kept in aliquots at −80°C until used for viral transduction.

### Hematopoietic stem cell enrichment and retroviral transduction

Bone marrow was extracted from the *femur* and *tibia* of mice 48 h after i.p. injection of 150 mg/kg of 5-fluorouracil (Mayne Pharma Pic, Warwickshire, UK). Bone marrow cells were enriched for hematopoietic progenitors and stem cells by negative selection using the StemSep kit (StemCell Technologies Inc., Vancouver, BC, Canada) according to the manufacturer's specifications. Cells were cultured for 24 hours in OPTIMEM (Invitrogen) supplemented with 10% FCS, 2 mM L-glutamine, 1 mM sodium pyruvate, 50 U/ml penicillin, 50 µg/ml streptomycin, IL-6, IL-3 and SCF containing supernatants [40]. The hematopoietic stem cells were then co-transduced by two rounds of spin infection with combinations of GFP, YFP, BCL-X_L_-GFP, BCL-2-GFP, FLIP_L_-GFP and MYC-YFP in the presence of 8 µg/ml polybrene (Sigma, St Louis, MO, USA). This procedure was repeated for three consecutive days and the cells were then cultured for another three days. The percentage of GFP and YFP positive cells were measured by flow cytometry prior to transplantation. Non-transduced cells were sometimes used to dilute GFP+/YFP+ cells to frequencies below 5%. Transplantation was performed by intravenous injection of 1×10^6^ cells into lethally irradiated (800rad for BALB/c or 960 rad for DBA/2) syngeneic recipient mice. The mice were given 8 µg/ml of doxycycline (Doxyferm; Merckle GmbH, Blaubeuren, Germany) in the drinking water and kept in filter top cages.

### Analysis of mice

Mice were monitored three times a week for signs of disease by palpation and observation and were judged as terminally ill when they displayed signs of paralysis in limbs or persistently hunched posture and slow movements. Mononuclear cell suspensions of spleen, thymus, liver, bone marrow and lymph nodes were obtained by passing tissues through nylon mesh cell strainers (BD Biosciences, Bedford, MA, USA). Tissues (lymph node, spleen, thymus, liver, kidney, heart, lung, bone) from the mice were fixed in buffered 4% formaldehyde.

### Flow cytometry analysis

Single cell suspensions from spleen, thymus, femoral bone marrow and liver were incubated with mAbs including anti-CD4-APC, anti-Gr1-APC, anti-CD19-bio, and anti-CD71-bio (BD PharMingen San Diego CA, USA). Pacific blue-labeled anti-CD8 (clone 2.43), anti-CD11b (clone M1/70.15) anti-Ter119 and anti-IgM (clone M41) MAb were prepared in our laboratory according to standard procedures. Biotinylated samples were then incubated with streptavidin-APC (BD PharMingen) before being analyzed on a Cyan ADP flow cytometer (Beckman Coulter, Inc., Fullerton, CA, USA). EGFP and EYFP were detected using 510/21-nm and 550/30-nm bandpass filters separated by a dichroic 525-nm mirror. Flow cytometric data were analyzed with FlowJo (Tree Star, Inc., Ashland, OR, USA).

## Results

### Transduction of hematopoietic stem cells with retroviruses expressing MYC alone or together with the anti-apoptotic genes BCL-X_L_ or FLIP_L_

**Figure 1:**
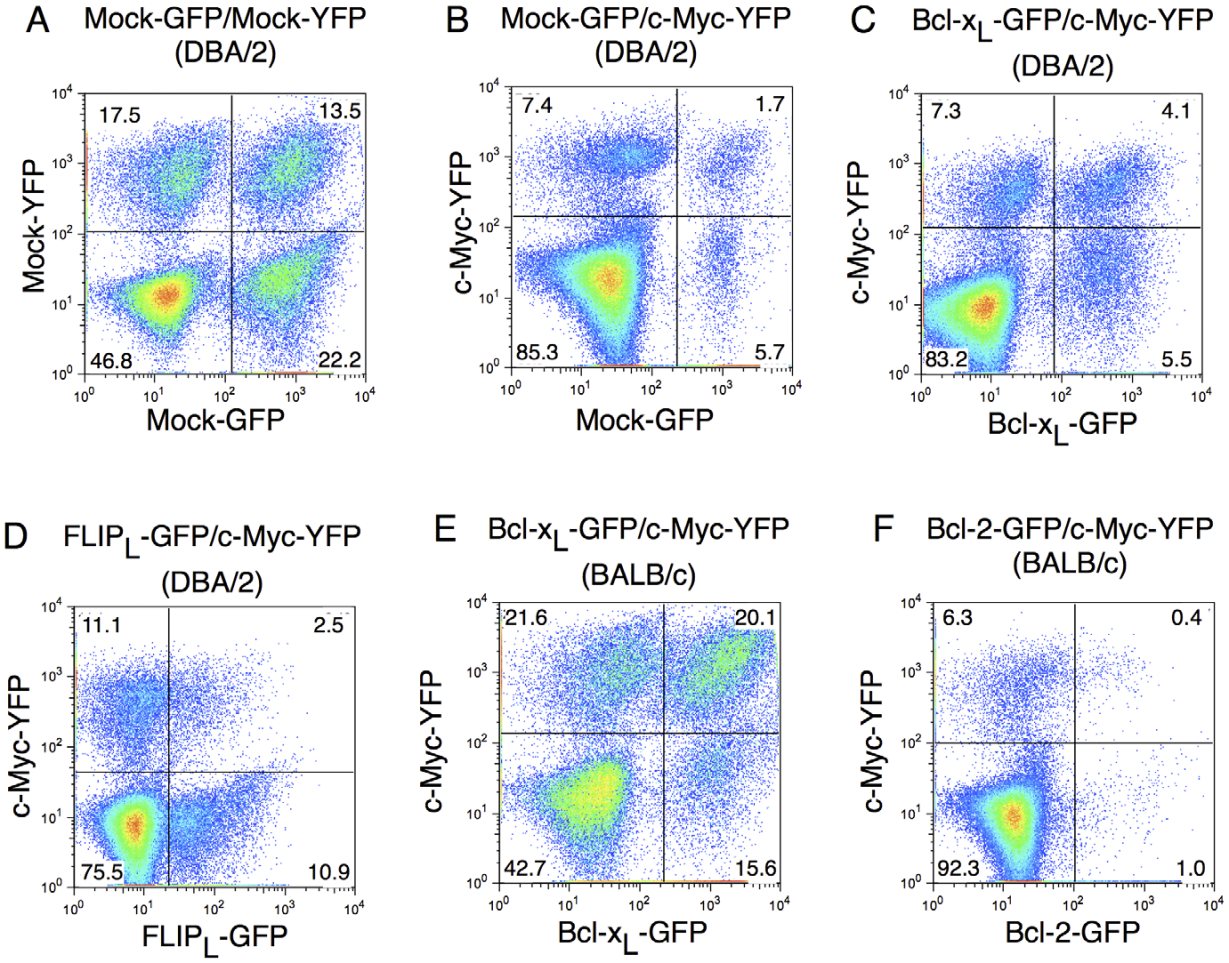
Expression of the various combinations of genes in Lin^−^ cells prior to transplantation. FACS analysis of expression of YFP and GFP in HSC prior to transplantation in HSC derived from (A) DBA/2 mice transduced with Mock-GFP and Mock-YFP, (B) DBA/2 mice transduced with Mock-GFP and MYC-YFP, (C) DBA/2 mice transduced with BCL-X_L_-GFP and MYC-YFP, (D) DBA/2 mice transduced with FLIP_L_-GFP and MYC-YFP, (E) BALB/c mice transduced with BCL-X_L_-GFP or MYC-YFP and (F) BALB/c mice transduced with BCL-2-GFP and MYC-YFP. The percentage of cells expressing the genes in each quadrant, gated on PI negative cells, is indicated.

To study the effects of anti-apoptotic gene products regulating the intrinsic and extrinsic apoptosis pathway, respectively, on MYC-driven transformation of hematopoietic stem cells, murine lineage-negative (Lin^−^) hematopoietic stem cells (HSC) were enriched from bone marrow and transduced with replication incompetent retroviral expression vectors, followed by transplantation to lethally irradiated syngeneic recipient mice. Expression of the gene of interest is in this system driven by the retroviral LTR promoter that gives expression in all lineages of hematopoietic cells [37], [41]. The gene of interest is followed by an internal ribosomal entry site (IRES) and GFP or YFP reporter genes, resulting in the production of a bicistronic mRNA. HSC were transduced with combinations of two different retroviruses, one carrying MYC-YFP and the other either BCL-X_L_ (regulating the intrinsic apoptotic pathway) or FLIP_L_ (regulating the extrinsic pathway) linked to GFP. As controls the same GFP and YFP vectors lacking other genes were used. Previously, using vectors expressing GFP or YFP, we have shown that this results in a near to random frequency of single and double expressing hematopoietic cells after transduction and transplantation [37]. This experimental approach allows for a competitive repopulation strategy, whereby the effects of overexpression of two genes of interest, either alone or in combination can be studied *in vivo*. The expression of YFP and GFP as determined by flow cytometry after transduction of various combinations of MYC, BCL-X_L_, FLIP_L_ or control vector into Lin^−^ bone marrow cells prior to transplantation is shown in Figure 1. Western blot analysis confirmed that MYC, BCL-X_L_ and FLIP_L_ were all expressed in cells transduced with the respective retroviral constructs (Figure S1A).

### Inhibition of the intrinsic but not the extrinsic pathway of apoptosis accelerates MYC-driven tumorigenesis and shortens survival

**Figure 2:**
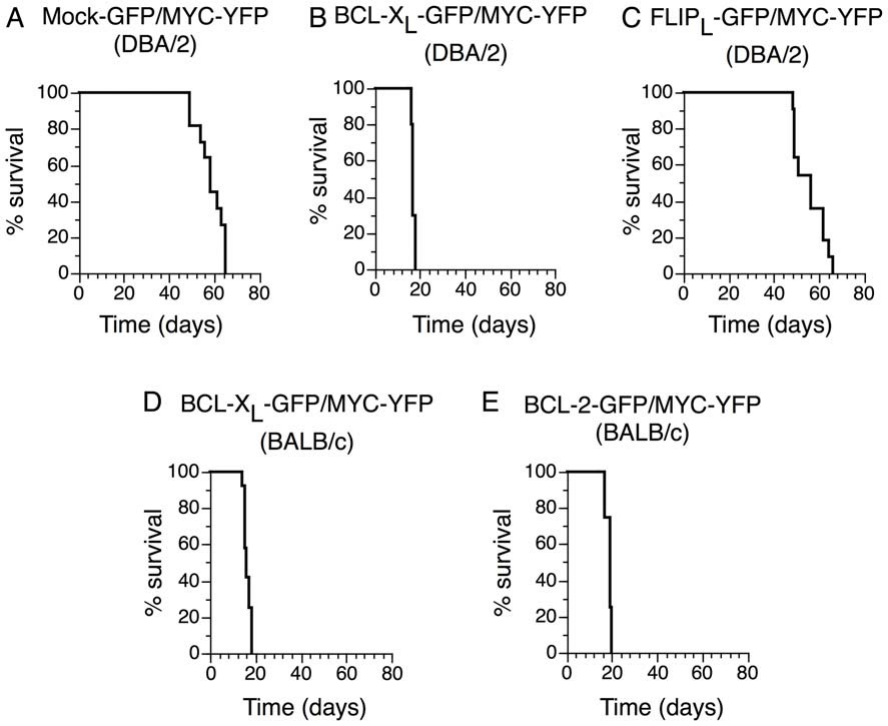
BCL-X_L_ and BCL-2 but not FLIP_L_ accelerate Myc-induced hematopoietic tumorigenesis. Kaplan-Meier survival analysis of mice transplanted with HSC over-expressing (A) Mock-GFP/MYC-YFP in DBA/2, (B) BCL-X_L_ -GFP/MYC-YFP in DBA/2, (C) FLIP_L_-GFP/MYC-YFP in DBA/2, (D) BCL-X_L_-GFP/MYC-YFP in BALB/c and (E) BCL-2-GFP/MYC-YFP in BALB/c. Mice were monitored daily for tumors or signs of paralysis and killed if showing signs of sickness. The percentage of mice surviving at daily intervals is shown.

Transplantation of Lin- bon marrow cells transduced with Mock-GFP/MYC-YFP resulted in reconstitution of myeloid, erythroid and lymphoid cells (Figure S2) but 7–9 weeks after transplantation, mice suddenly started to display signs of disease. Early signs of disease were ruffled fur, wasting and slow movements that rapidly developed into hind limb paralysis within two days. In some cases, also front leg paralysis was detected, and these animals showed mediastinal tumors invading the spine. Necropsy of moribund animals showed splenomegaly, grossly enlarged livers, non-coagulating blood, spotted lungs and in many animals also enlarged lymph nodes and thymus. This was in sharp contrast to mice receiving HSC transduced with Mock-GFP/Mock-YFP that remained healthy during the whole study period of 6 months (data not shown), despite higher frequencies of retroviral transduction (Figure 1A). The median survival after transplantation of Mock-GFP/MYC-YFP recipient mice was 56 days after transplantation (Figure 2A). This is in good agreement with previous reports on transplantation of MYC-transduced HSC into irradiated mice [11], [19], [42], [43].

In order to examine the influence of the intrinsic pathway of apoptosis during MYC-induced malignant transformation HSC were transduced with a combination of MYC and BCL-X_L_ (Figure 1C). BCL-X_L_/MYC recipient mice rapidly became moribund and had a median survival of only 17 days (Figure 2A, B and Table 1). BCL-X_L_ therefore significantly accelerated MYC-induced tumorigenesis. BCL-X_L_/MYC mice displayed similar signs of disease as Mock/MYC mice, namely ruffled fur and slow movements that developed into hind limb paralysis. Necropsy of moribund BCL-X_L_/MYC mice showed that tumors were disseminated throughout the hematopoietic tissues with extensive involvement of spleen and bone marrow and tumor infiltration of the liver. We further tested whether over-expression of FLIP_L_, a cellular inhibitor of death receptor-mediated apoptosis, would influence MYC-driven malignant transformation of hematopoietic cells. HSC were co-transduced with retroviral expression vectors containing MYC-YFP and FLIP_L_-GFP (Figure 1D). Surprisingly, FLIP_L_/MYC mice displayed similar signs of disease and with a similar kinetics as Mock/MYC mice (Figure 2A and 2C). All FLIP_L_/MYC mice died or were euthanized due to severe signs of sickness within 9 weeks with a 58 days median survival time for FLIP_L_/MYC to be compared with 56 days for Mock/MYC mice. The difference in survival time between Mock/MYC mice and FLIP_L_/MYC was not statistically significant whereas the difference in survival times between Mock/MYC and BCL-X_L_/MYC mice was highly significant (Table 1). Necropsy of moribund FLIP_L_/MYC animals showed similar features as Mock/MYC mice, including splenomegaly, hepatomegaly, non-coagulating blood, spotted lungs and in many animals also enlarged lymph nodes and thymus.

**Table 1:** Statistical analysis of survival data after transplantation of hematopoietic stem cells expressing MYC, BCL-X_L_, BCL-2 and/or FLIP_L_.

	Mock-GFP/MYC-YFP (DBA/2)	FLIP_L_-GFP/MYC-YFP (DBA/2)	BCL-X_L_-GFP/MYC-YFP (DBA/2)	BCL-X_L_-GFP/MYC-YFP (BALB/c)
FLIP_L_-GFP/MYC-YFP (DBA/2)	NS* p = 0.5077	-		
BCL-X_L_-GFP/MYC-YFP (DBA/2)	S p<0.0001	S p<0.0001	-	
BCL-X_L_-GFP/MYC-YFP (BALB/c)	S p<0.0001	S p<0.0001	NS p = 0.2156	-
BCL-2-GFP/MYC-YFP (BALB/c)	S p<0.0001	S p<0.0001	S p = 0.0004	S P<0.0001

Statistical analysis of the survival data presented in Figure 2.

* Log-rank (Mantel-Cox) test. NS = non significant. S = significant. P-value indicated below.

**Figure 3:**
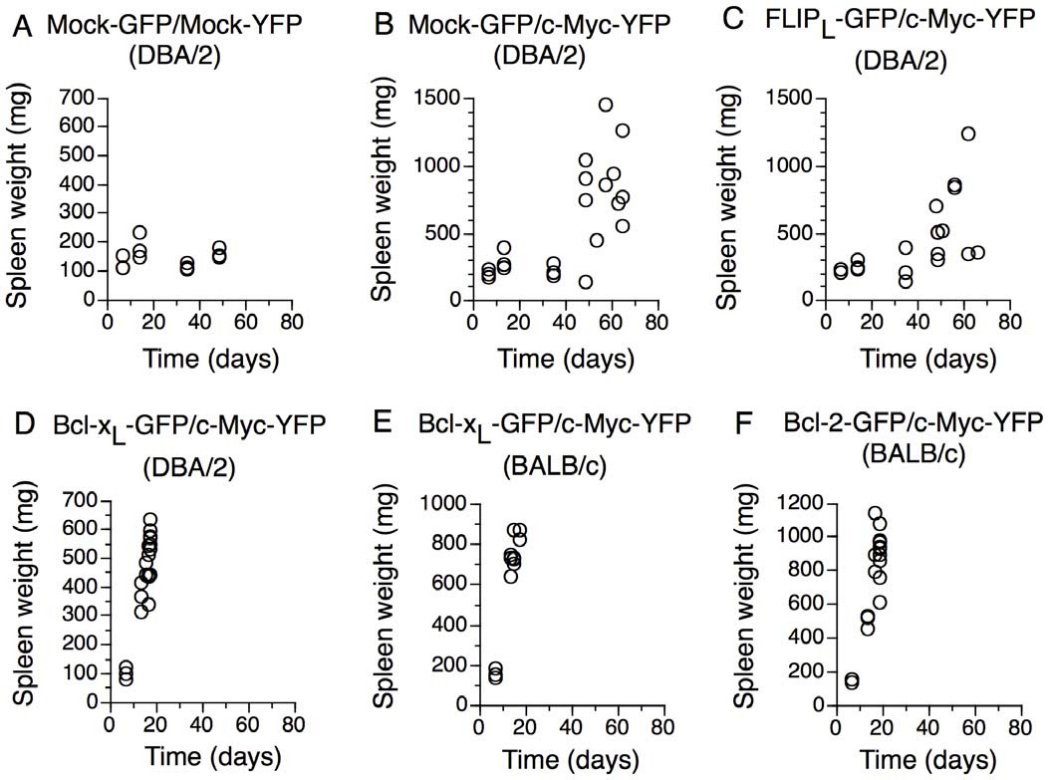
Over-expression of BCL-X_L_ and BCL-2 accelerate Myc-induced splenomegaly whereas co-expression of FLIP_L_ does not influence MYC-induced splenomegaly. Spleen weight of animals transplanted with HSC expressing (A) Mock-GFP/Mock-YFP into DBA/2 mice, (B) Mock-GFP/MYC-YFP into DBA/2 mice, (C) FLIP_L_-GFP/MYC-YFP into DBA/2 mice, (D) BCL-X_L_-GFP/MYC-YFP into DBA2 mice, (E) BCL-X_L_-GFP/MYC-YFP into BALB/c mice and (F) BCL-2-GFP/MYC-YFP into BALB/c. Spleens of mice at control time points (7 days, 14 days, 35 days and 49 days after transplantation) and spleens of all moribund mice were weighed. Each dot represents one mouse.

Spleen weights of recipient mice (showing no signs of disease) were recorded at 7, 14, 35 and 49 days after transplantation. Figure 3 shows spleen weights at these time points as well as spleen weights of all moribund mice. Mock-GFP/Mock-YFP recipient mice did not show signs of splenomegaly at any time although they had somewhat larger spleens 14 days after transplantation likely due to intensive reconstitution after irradiation and transplantation (Figure 3A). Mock-GFP/MYC-YFP mice only had slightly increased spleen sizes at early time points up to 5 weeks after transplantation (mean weight: 236 mg compared to 138 mg for Mock-GFP/Mock-YFP recipients). However, at later time points (+7 weeks), Mock-GFP/MYC-YFP recipient mice developed severe splenomegaly (mean weight: 816 mg) (Figure 3B). Co-expression of BCL-X_L_ together with MYC resulted in accelerated development of severe splenomegaly in all animals 2 weeks after transplantation with mean values for moribund animals at 504 mg compared to 180 mg in control Mock/Mock animals and 260 mg in control Mock/MYC mice at 2 weeks after transplantation (Figure 3A, B and D).

As in Mock/Myc mice, FLIP_L_/MYC mice appeared to have a latency period before development of splenomegaly (average spleen weight at 5 weeks after transplantation, 237 mg for FLIP_L_/MYC compared to 236 mg for Mock/MYC and 138 mg for Mock/Mock recipient mice) (Figure 3). Moribund FLIP_L_/MYC mice at later time points (+7 weeks) had grossly enlarged spleens (average 579 mg), to a similar extent as Mock/MYC mice (average 816 mg).

To ensure that the FLIP_L_ construct was functionally active, Fas-sensitive A20 cells were transduced with the FLIP_L_-GFP construct and thereafter incubated with or without agonistic anti-Fas mAb for 20 hours and thereafter analyzed by flow cytometry. Figure S1B shows that FLIP_L_ efficiently inhibited Fas-induced apoptosis in this system. This suggested that inhibition of the intrinsic but not the extrinsic pathway of apoptosis synergized with MYC to promote tumorigenesis of HSC.

### Inhibition of the intrinsic but not the extrinsic pathway of apoptosis drives MYC-driven tumorigenesis towards acute myeloid leukemia

**Figure 4:**
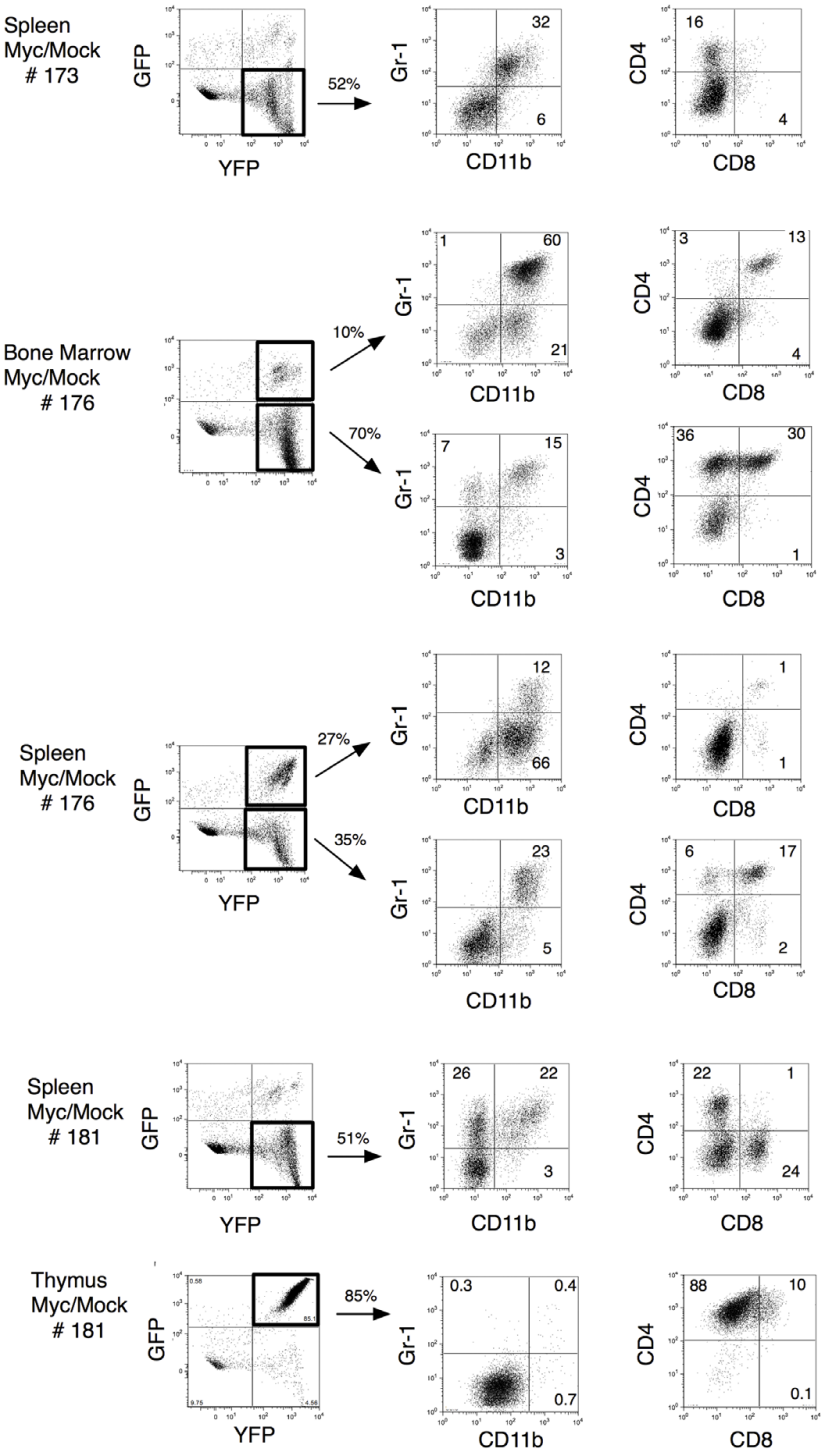
Overexpression of MYC in HSC induces both myeloid and lymphoid leukemia. Flow cytometry analysis of bone marrow and spleen cells from two individual moribund Mock/MYC mice (indicated with # in the left). In the middle, percentage of GFP^+^YFP^+^ (Mock/MYC expressing) cells or GFP^−^YFP^+^ (MYC expressing) cells are indicated. These cells were further characterized with anti-CD11b, anti-Gr1, anti-CD4, anti-CD8, anti-CD19, anti-IgM anti-CD71 and anti-Ter119. The percentage of cells in each quadrant is indicated.

We next investigated the spectrum of tumors generated by expression of MYC alone and by coexpression of MYC together with BCL-X_L_ or FLIP_L_, respectively, using flow cytometry. Single cell suspensions from femoral bone marrow, spleen, thymus, liver and (in a few individual mice) enlarged lymph nodes of mesenteric or cervical origin were stained with the myeloid markers anti-CD11b and anti-Gr1, the T-cell markers anti-CD4 and anti-CD8, the B-cell markers anti-CD19 and anti-IgM, and the erythroid markers anti-CD71 and Ter119. Mock/Mock animals all showed similar staining patterns with normal frequencies of myeloid as well as lymphoid cells (Table S1a). Each Mock/MYC animal displayed an individual setup of tumors of both myeloid lineages as well as tumors of T-cell lineage origins (Figure 4, Table S1b). Moreover, each Mock/MYC animal typically displayed diverse tumors in different cellular compartments. Bone marrow often contained tumors of myeloid origin whereas thymus and lymph nodes (if enlarged) contained T-cell tumors. Spleen and infiltrating cells of the liver frequently showed a mixed population of both myeloid and lymphoid origin with increased numbers in separate animals of both T-cells (in most cases CD4^+^, but also individuals with increased numbers of CD8^+^ and CD4^+^CD8^+^ cells) and often myeloid cells (Gr1^+^CD11b^+^, CD11b^+^ and/or Gr1^+^). Analyses of spleen cells from a few representative Mock/MYC mice are shown in Figure 4. Individual animals could have increased numbers of both MYC (GFP^−^YFP^+^) and Mock/MYC (GFP^+^YFP^+^) cells as exemplified by MYC/Mock #176 spleen (Figure 4) where the GFP^+^YFP^+^ subpopulation was mainly CD11b^+^ and the GFP^−^YFP^+^ subpopulation could be further divided into a Gr1^+^CD11b^+^ and a CD4^+^CD8^+^ population, indicating that these tumors were of oligoclonal nature.

**Figure 5:**
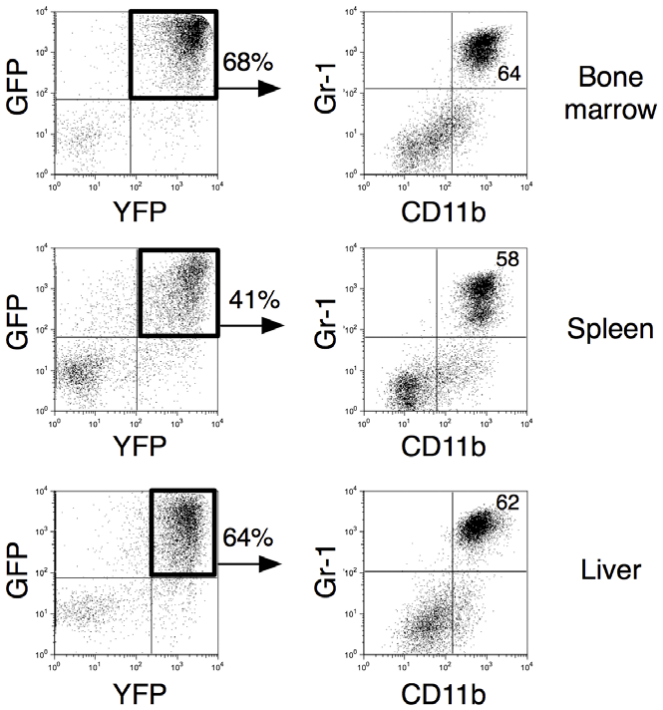
Overexpression of BCL-X_L_-GFP/MYC-YFP induces AML. Flow cytometry analysis of bone marrow (top), spleen (middle) and liver (bottom) cells from one representative moribund BCL-X_L_-GFP/MYC-YFP mouse. Single cell suspensions from femoral bone marrow, spleen and liver were stained with anti-CD11b and anti-Gr1. The percentage of dominating cells is indicated.

In contrast, the vastly dominating cell type among BCL-X_L_-GFP/MYC-YFP expressing cells were myeloid CD11b^+^Gr1^+^ cells, which accounted for around 50% of the cells in bone marrow and spleen (Table S1d and Figure 5). Also the livers of moribund mice were heavily infiltrated with BCL-X_L_-GFP/MYC-YFP expressing myeloid CD11b^+^Gr1^+^ cells (Figure 5). Mice transplanted with MYC/BCL-X_L_ had a pronounced leukocytosis compared to Mock/Mock transplanted mice (Figure S3). A differential count of the blood confirmed that approximately 50% of cells in the MYC/BCL-X_L_ mice were blasts (Figure S3 and Table S2). These results indicate that BCL-X_L_ drives MYC-driven tumorigenesis towards acute myeloid leukemia. These results differ from a report by Luo et al [11] that suggested that expression of MYC alone in bone marrow cells transplanted into recipient hosts give rise to AML, while coexpression of MYC and BCL-2 gave rise to a mixture of AML and pre-B acute lymphoid leukemia in BALB/c mice (see Discussion). In order to generalize our finding to other anti-apoptotic members of the BCL-2 family and also to other strains of mice, MYC was over expressed together with BCL-X_L_ or BCL-2 in HSC of BALB/c mice followed by transplantation (Figure 1E-F). Mice transplanted with this combination rapidly developed a similar AML-like disease as did DBA/2 mice transplanted with HSC over expressing BCL-X_L_ and MYC. Mean survival time was 16 days for BCL-X_L_/MYC BALB/c mice (Figure 2D) and 19 days for BCL-2/MYC BALB/c mice (Figure 2E). This minor difference in survival time between BCL-X_L_/MYC and BCL-2/MYC mice was statistically significant but is most likely a reflection of the lower proportion of BCL-2/MYC double expressing cells among the transplanted cells (Table 1, Figure 1). Co-expression of BCL-X_L_ or BCL-2 together with MYC resulted in splenomegaly in all animals with mean values for moribund animals at 757 mg for BCL-X_L_/MYC BALB/c (Figure 3E) and 899 mg for BCL-2/MYC BALB/c mice (Figure 3F). All tumors predominantly expressed the granulocyte markers Gr1 and CD11b and were GFP^+^/YFP^+^ (Figure 5, Table S1e-f), thus confirming the results from the DBA/2 mice. For MYC alone, similar results concerning the kinetics of tumor development and the frequencies of myeloid versus lymphoid tumor cells were obtained using BALB/c mice (data not shown).

As in Mock/MYC mice, FLIP_L_/MYC mice primarily developed tumors of myeloid (Gr1^+^CD11b^+^, CD11b^+^ and/or Gr1^+^) or T-cell lineage origin (CD4^+^). FLIP_L_/MYC spleen and infiltrating cells of the liver frequently showed a mixed population of both myeloid and lymphoid origin as in Mock/MYC mice (Table S1b-c). One notable difference to cells expressing MYC alone was that the double positive CD4^+^CD8^+^ lymphoid tumor cells population was diminished among FLIP_L_/MYC-expressing cells in favor of the CD4+ population (Table S1c). This might suggest that FLIP_L_ plays a role in the selection of MYC-induced lymphoid but not of myeloid tumor cells in this system.

Immunohistochemical analyses confirmed and extended the cytometrical results. Sections of spleen and liver were stained with Abs against the T-cell markers CD45R and CD3 and the myeloid marker myeloperoxidase (MPO) (See Materials and Methods S1). Extensive staining with CD3 and MPO could be observed in spleen and liver of Mock/MYC recipient mice. In liver, CD3^+^ cells were predominantly distributed as perivascular “cuffs” in contrast to a more diffuse distribution of myeloid cells. Splenic architecture was heavily distorted with an expanded red pulp, immunohistochemically dominated by CD3^+^ and MPO^+^ cells (Figure S4). No infiltration could be detected in livers of Mock/Mock recipient mice and spleens showed a normal architecture (Figure S5). In MYC/BCL-X_L_ or MYC/BCL-2 mice tumor cells in spleen and liver expressed the myeloid marker MPO but not the T-cell markers (Figure S6 and S7). Immunohistochemical stainings of tumor cells in FLIP_L_/MYC mice showed similar staining patters as Mock/MYC (Figure S8).

Tumor cell lines could be established from all moribund Mock/MYC, MYC/BCL-X_L_, MYC/BCL-2 and MYC/FLIP_L_ recipient mice. These cells were further characterized by flow cytometry and all MYC-YFP^+^ appeared with similar phenotypes as the primary tumors (data not shown).

**Figure 6:**
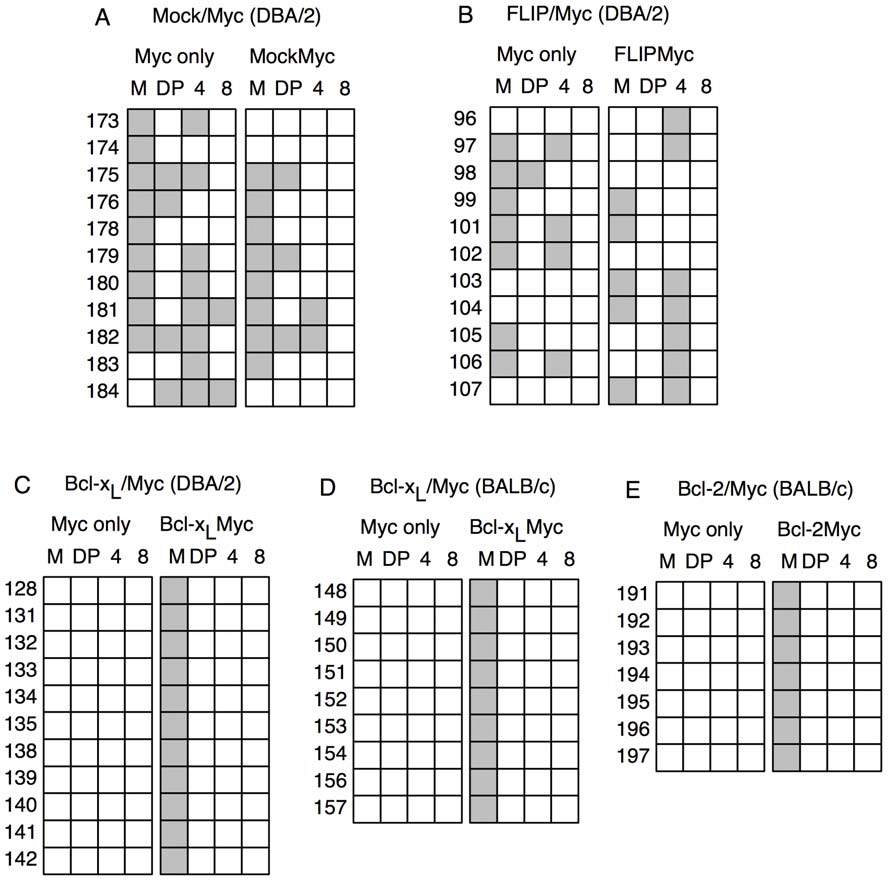
Tumor phenotype in Mock/MYC, FLIP_L_/MYC, BCL-X_L_/MYC and BCL-2/MYC recipient mice. Summary of flow cytometry analysis of spleen cells from moribund mice transplanted with HSC expressing (A) Mock-GFP/MYC-YFP into DBA/2 mice, (B) FLIP_L_-GFP/MYC-YFP into DBA/2 mice, (C) BCL-X_L_-GFP/MYC-YFP into DBA2 mice, (D) BCL-X_L_-GFP/MYC-YFP into BALB/c mice and (E) BCL-2-GFP/MYC-YFP into BALB/c mice. Left panel specify tumors expressing MYC-YFP only and right panel specify cells expressing MYC-YFP together with either Mock-GFP (A), FLIP_L_-GFP (B), BCL-X_L_-GFP (C and D) or BCL-2-GFP (E). Filled box indicate tumor phenotype. M indicates tumors of myeloid lineage, DP indicate CD4^+^CD8^+^ lymphoid tumors, 4 indicate CD4^+^ lymphoid tumors and 8 indicate CD8^+^ lymphoid tumors. Numbers correspond to identity of individual animal.

These results are summarized in Figure 6 and suggest that Mock/MYC mice developed myeloid leukemia and lymphoblastic T-cell lymphoma. Coexpression of MYC and BCL-X_L_ or BCL-2 strongly favored tumor development towards myeloid leukemia, whereas coexpression of MYC and FLIP_L_ did not affect the distribution of myeloid versus lymphoid tumor cells, but changed the ratio between single CD4^+^ and double positive CD4^+^CD8^+^ T-cell lymphomas.

### Early expansion of myeloid leukemic cells in MYC and MYC/BCL-X_L_ expressing mice at the expense of other hematopoietic cell populations

In order to follow leukemia/lymphoma development kinetically and to gain insight into how different blood cell populations were affected by MYC and MYC/BCL-X_L_ expression, hematopoietic development was studied in detail at 7, 14 and 35 days after transplantation of Mock/MYC- or MYC/BCL-X_L_-transduced HSC into recipient mice. Figure S2 (middle columns, top row) shows that MYC-YFP-expressing cells dominated among cells in bone marrow and spleen already at day 7. In bone marrow and spleen, MYC-YFP-expressing cells constituted about 50% of all cells. This figure was rather constant between 7 and 35 days after transplantation. At the time of transplantation, only about 6% of the cells expressed MYC-YFP, indicating that these cells underwent a rapid expansion to reach high steady-state levels *in vivo*. Mock-GFP/Mock-YFP-expressing cells, on the other hand, did not expand and were kept at constant relative numbers reflecting the proportions of transduced cells before transplantation (Figure S2, left columns, top row). BCL-X_L_-GFP/MYC-YFP expressing cells in bone marrow expanded from about 20% of the cells at day 7 to 75% at day 14 after transplantation. In spleen, BCL-X_L_-GFP/MYC-YFP expressing cells expanded from 20% at day 7 to 40% at day 14 (Figure S2, right columns, top row).

Next, the phenotypes of cells expressing Mock-GFP, MYC-YFP and BCL-X_L_-GFP/MYC-YFP were analyzed. In figure S2 (middle panels) it can be seen that the dominating cell type among expanded MYC expressing cells was the myeloid CD11b^+^Gr1^+^ cell population, which accounted for around 60% of the cells in the bone marrow and 20–30% in spleen already at day 7 and thereafter. Corresponding figure for Mock-expressing cells was considerably lower. MYC expression was readily detectable in pre-B, B, CD4^+^ and CD8^+^ T cells and in erythroid Ter119^+^CD71^hi^ and Ter119^+^CD71^lo^ cells but did not result in their expansion. A tendency towards expansion of CD19^+^IgM^−^ and CD8^+^ T cells could be recorded among MYC-expressing cells in the spleen at day 35. BCL-X_L_-GFP/MYC-YFP expressing cells could be found in pre-B, B, CD4^+^, CD8^+^ T cells and in erythroid Ter119^+^CD71^hi^ and Ter119^+^CD71^lo^ cells, in particular at day 14 (Figure S2, right panels). However, the vastly dominating cell type among expanded BCL-X_L_-GFP/MYC-YFP expressing cells already at day 7 were myeloid CD11b^+^Gr1^+^ cells, which accounted for around 60% of the cells in bone marrow and spleen at day 14.

Overall, these results indicate that the myeloid CD11b^+^Gr1^+^ population dominated at early time points both in mice transplanted with MYC-YFP- and BCL-X_L_-GFP/MYC-YFP-expressing HSCs. The expansion of other cell populations lagged behind and was only evident in MYC-expressing mice at day 35, a time point where the MYC/BCL-X_L_ mice had already died from AML.

### MYC- and MYC/BCL-X_L_-expressing CD11b^+^Gr1^+^, pre-B and B cells become blast transformed early after transplantation

**Figure 7:**
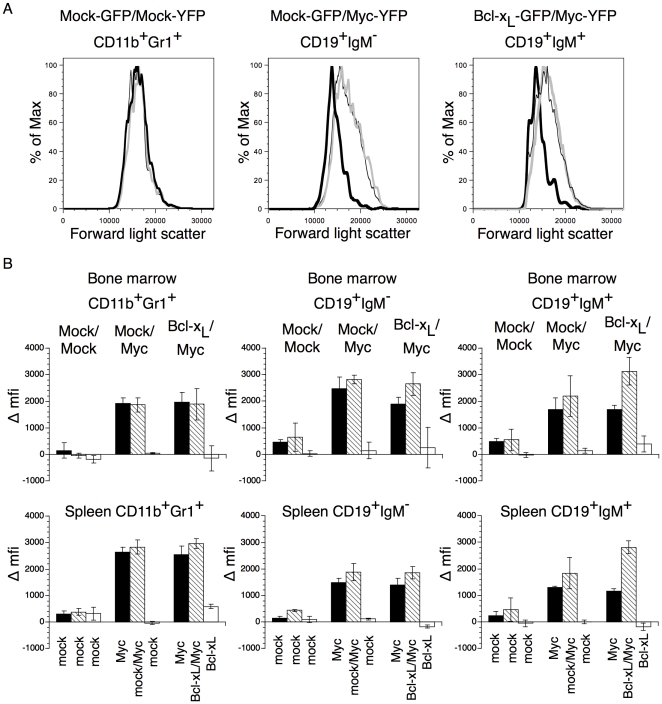
Blast formation of Mock-GFP/Mock-YFP, Mock-GFP/MYC-YFP or BCL-X_L_-GFP/MYC-YFP bone marrow and spleen cells. (A) Forward light scatter of CD11b^+^Gr1^+^ bone marrow cells from Mock-GFP/Mock-YFP, Mock-GFP/MYC-YFP and BCL-X_L_-GFP/MYC-YFP recipient mice 2 weeks after transplantation. Grey thick lines indicate GFP^−^YFP^+^ cells, thick black lines indicate GFP^+^YFP^−^ cells and thin lines indicate GFP^+^YFP^+^ cells. (B) Difference in mean fluorescence intensity (Δmfi) in forward scatter of CD11b^+^Gr1^+^ (left), CD19^+^IgM^−^ (middle) and CD19^+^IgM^+^ (right) cells in bone marrow (top) and spleen (bottom) between GFP^−^YFP^−^ (non-transduced cells) and GFP^−^YFP^+^ (black bars), GFP^+^YFP^+^ (striped bars) or GFP^+^YFP^−^ (white bars) cells are shown. Values indicate means of three individual mice and error bars indicate 1 SD.

To study activation and blast transformation of myeloid and B lymphoid cells, differences in mean fluorescence intensity (Δmfi) in forward scatter were analyzed in CD11b^+^Gr1^+^, CD19^+^IgM^−^ and CD19^+^IgM^+^ cells from bone marrow and spleen 14 days after transplantation. BCL-X_L_/MYC and MYC expressing cells of all these phenotypes were clearly blast transformed compared to non-transduced cells, cells expressing Mock-GFP or cells expressing only BCL-X_L_-GFP within the same animal (Figure 7, A–B middle and right panels). Similar results were obtained at 7 days and 35 days after transplantation (data not shown). For CD19+IgM+B cells, increased cell size was noted for MYC/BCL-X_L_ cells compared to cells expressing MYC only, perhaps indicating increased survival of such cells in the presence of BCL-X_L_. It was, however, mainly MYC- and MYC/BCL-X_L_-expressing myeloid CD11b^+^Gr-1^+^ cells that expanded and rapidly reached elevated steady-state levels (Figure S2). In summary, mice transplanted with MYC- or MYC/BCL-X_L_-expressing cells, contained large number of pre-malignant MYC-expressing cells of various lineages. These populations remained at steady-state levels until about 6–7 weeks after transplantation in MYC-expressing mice, when exponential growth of some of these cell populations suddenly ensued. At this time point MYC/BCL-X_L_-expressing mice had died from AML.

## Discussion

Several lines of evidence from the human disease and mouse models have implicated the *MYC* oncogene in development of AML [3], [4], [8], [10], [11], [19], [42], [43], [44], [45], [46]. However, deregulated MYC expression has also been shown to induce the two major pathways of apoptosis, the intrinsic and the extrinsic (death receptor) pathways [15], [20], [23], [34], [35], [47]. This anti-tumorigenic property of MYC needs to be overcome for tumor development to occur, and mutations or deregulated expression of components of both the intrinsic and extrinsic pathways have been documented in AML [36], although the relative contribution of these pathways for the disease has not been investigated systematically. In order to explore the role of these two pathways of apoptosis during MYC-induced AML, we have overexpressed MYC in HSC, the tumor-initiating cell of AML, either alone or together with cellular inhibitors of the intrinsic or the extrinsic pathways of apoptosis of particular relevance for AML; BCL-X_L_, BCL-2 and FLIP_L_. The transduced HSC were transplanted to lethally irradiated syngeneic recipient mice after which tumor development was monitored.

Our results show that mice reconstituted with HSC overexpressing MYC developed myeloid leukemia and T cell lymphoma within 9 weeks, in agreement with previous reports [11], [19], [42], [43]. Importantly, coexpression with BCL-X_L_ or BLC-2 strongly accelerated MYC-induced tumorigenesis and all mice died or were moribund within 2 weeks after transplantation. Co-expression of MYC with BCL-X_L_ or BCL-2 also influenced the tumor phenotype; Rather than a mixed phenotype of myeloid and T lymphoid tumors, development of an aggressive AML-like disease was favored (Figure 6). The synergistic effect of MYC and BCL-X_L_ or BCL-2 overexpression on tumor development observed here is in agreement with previous observations in transgenic Eμ-MYC/BCL-2-induced B lymphoma [17], [18]. However, although using an AML mouse model based on transplantation of transduced bone marrow cells into irradiated recipient hosts similar to this study, Luo et al. [11] reported that BCL-2 did not accelerate MYC-induced AML, but rather stimulated development of pre-B acute lymphoid leukemia (ALL). We could not reproduce the results of Luo et al in our model using the same genes and mouse strain (see further discussion below). Our detailed studies of the kinetics of tumor development showed that overexpression of MYC, either alone or together with BCL-X_L_ or BCL-2, rapidly resulted in blast transformation of various hematopoietic cell types, including myeloid and lymphoid cells. However, only myeloid cells expanded, and in the case of MYC expression they rapidly reached a steady-state level that was kept until about 7–9 weeks after transplantation, when suddenly a fatal expansion ensued. In the case of MYC and BCL-X_L_ or BCL-2 co-expression, myeloid cells expanded exponentially already within a few days after transplantation. Our results indicate that different hematopoietic cell lineages are differentially permissive for oncogenic transformation. It is likely that an early expansion of the MYC-transformed myeloid leukemia cells is counteracted by MYC-induced apoptosis that is overcome by BCL-X_L_ or BCL-2 expression, resulting in an almost immediate tumor transformation of myeloid CD11b/Gr1-positive cells, subsequently causing AML. Our results are in line with a recently published study by Beverly and Varmus [19] in which MYC was overexpressed together with each of the 6 known anti-apoptotic members of the BCL-2 family in bone marrow cells transplanted into irradiated hosts like here, all resulting in acceleration of MYC-induced AML-like disease. The reason for the discrepancy in relation to Luo et al. [11] is unclear, but may be due to differences in the purification of the retroviral target bone marrow cell population (see further discussion below).

In contrast to the effects of BCL-X_L_ and BCL-2 overexpression, FLIP_L_ although functional, did not enhance MYC-driven tumorigenesis, neither with respect to the kinetics nor the relative proportion of myeloid leukemia versus T cell lymphoma compared with MYC alone. This result was unexpected considering previous reports that Myc sensitizes cells, including hematopoietic cells, to death receptor-induced apoptosis [12], [13], [22], [23], [24], [25], [27], [35], [48], involving at least in part MYC-mediated repression of FLIP_L_ expression [34], [35]. There could be several different explanations to this result. First, it should be noted that all previous studies on the relation between MYC and FLIP_L_ has been performed in cell cultures in vitro, and may be less relevant for tumor development in vivo. Nevertheless, TRAIL-R deficiency has been shown to enhance MYC-induced lymphomagenesis in the Eμ-*myc* model [27]. However, recent studies suggest that MYC sensitizes death receptor signaling mainly by regulating BCL-2-family proteins of the intrinsic pathway and by suppressing NFκB [28], [49], which all are involved in crosstalk with the extrinsic pathway. Through this mechanism several pathways of MYC-induced apoptosis would be blocked by BCL-X_L_ and BCL-2, which may explain their particular importance in Myc-driven AML as shown here. Still, even if MYC mainly sensitizes the death receptor pathway via regulating BCL-2-family proteins and NFκB, we would expect overexpression of FLIP_L_ to have some impact if the death receptor pathway is important for suppressing AML development. Another possible explanation is that the death receptor pathway and its regulation by FLIP_L_ is important for certain MYC-induced tumors, such as Eμ-*myc* driven B cell lymphoma, but less important in the case of AML. One difference between MYC and MYC/FLIP_L_ mice was that the ratio between immature CD4^+^/CD8^+^ and mature CD4^+^ T-cell lymphoma was skewed towards CD4^+^ lymphoma. This might indicate that overexpression of FLIP_L_ favors differentiation of T-cell lymphoma cells or increases the survival of mature CD4^+^ T-cell lymphoma cells, but does not change the overall tumor burden. Previous studies of Myc-induced CD4^+^/CD8^+^ T–cell lymphoma in the CD2-MYC model in a *lpr* genetic background showed that deletion of Fas did not accelerate T-cell lymphoma development, although an increased population of CD4^+^ T-cells was found [50]. Smith et al. [45] reported that MYC-expressing CD4^+^ T cells in the VavP-Myc model was more prone to apoptosis than the corresponding CD4^+^/CD8^+^ T-cells, suggesting that FLIP_L_ might contribute to increased survival of this population in our model system, although this did not contribute to increased T-cell lymphoma over AML ratio. Any potential impact of FLIP_L_ on MYC-induced B lymphomagenesis seems to be masked in this system by the rapid development of AML and T-cell lymphoma. This conclusion is not incompatible with FLIP_L_ being important for Myc-driven development of other types of tumors, such as B lymphoma (26, 27), under other conditions.

Although MYC was over expressed in cells of all hematopoietic lineages and resulted in blast transformation, morbidity was primarily due to development of myeloid and/or T-cell tumors.

T-cell lymphomas were similar both in phenotype (CD4^+^ or CD4^+^/CD8^+^) and latency (7–10 weeks) to those of VavP-MYC17 mice [51]. VavP-Myc17 transgenic animals with high expression of MYC in nucleated cells of hematopoietic origin have been reported to mainly develop T-cell lymphomas [51] whereas transgenic animals with low expression of c-Myc instead mainly developed myeloid monocytic tumors and only 7% T-cell lymphomas [45], suggesting that different expression levels of MYC may provoke different tumor phenotypes. In our experimental system, we were unable to find a direct correlation between MYC expression levels and myeloid or lymphoid phenotypes, but we cannot completely rule out that MYC expression levels may influence the disease phenotype. Most reports where MYC is expressed broadly in hematopoietic cell types in mice, it seems to generate either myeloid leukemia, T lymphoma or a mix of these two tumor types like here, which may reflect different levels of MYC depending on expression systems used [11], [19], [42], [43], [45], [46], [51]. Retroviral expression of MYC in bone marrow cells like here seems more often to result in predominance of myeloid leukemia [11], [42], [43], possibly due to lower levels of MYC than in the Vav promoter system. Another possibility is that the tumor initiating MYC target cell(s) differ between the systems. This may also explain differences in MYC-induced tumor types between different retroviral transduction systems. For instance, Luo et al. [11], who observed AML in the absence of T-cell lymphoma, used unfractionated mononuclear bone marrow cells, whereas we transduced Lin^−^ bone marrow cells. In another transplantation model, overexpression of MYC resulted in development of aggressive pre-B cell lymphomas, with low penetrance and after long latency (>100 days) [21]. Here, fetal liver (E14.5) cells were used for retroviral transduction. Also other methodological aspects such as choice of retroviral backbone, the use of single or dual expression vectors or time of culture in vitro prior to transplantation may influence experimental outcome.

In conclusion, our results suggest that MYC-induced transformation of HSC accelerates and polarizes hematopoietic tumor development towards aggressive AML by coexpression of suppressors of the intrinsic but not the extrinsic pathway of apoptosis. Since all these players have been implicated in the development of human AML, these results may potentially be of relevance for further preclinical and clinical studies of this disease.

## Supporting Information

Figure S1 **Expression and functionality of retroviral constructs.** **A**. Analysis of proteins from MYC/BCL-X_L_ tumor cells and Fas-sensitive A20 cells transduced with MSCV-hFLIP_L_-IRES-GFP retroviral particles. **B**. Fas-sensitive A20 cells were transduced with MSCV-hFLIP_L_-IRES-GFP retroviral particles. Transduced or wt A20 cells were thereafter incubated with or without agonistic anti-Fas mAb (0.25 µg/ml of Jo2) for 20 hours and thereafter analyzed by flow cytometry using propidium iodide (PI) to discriminate between live and dead cells.
(TIF)

Figure S2 **Early hematopoietic development of DBA/2 mice reconstituted with Mock-GFP/Mock-YFP, Mock-GFP/MYC-YFP or BCL-X_L_-GFP/Myc-YFP expressing HSCs.** Phenotypic analysis of bone marrow (left) and spleen (right) at 7, 14 and 35 days after transplantation of Mock-GFP/Mock-YFP, Mock-GFP/MYC-YFP or BCL-X_L_-GFP/MYC-YFP expressing HSCs into DBA/2 mice was performed with FACS. The horizontal axis indicates days after transplantation and the vertical axis represents percentage of cells expressing the marker indicated to the left. Black triangles: GFP^+^YFP^−^ cells, black squares; GFP^−^YFP^+^ cells and white circles; GFP^+^YFP^+^ cells.
(TIF)

Figure S3 **Appearance of leukemic blasts in the blood of MYC/BCL-X_L_ recipient mice.** Blood smears stained with May-Grunewald-Giemsa. Blood smear from a Mock/Mock mouse shows a normal picture with few leukocytes (A) as compared to the leukocytosis seen in MYC/BCL-X_L_ mice (B) (primary magnification 20×). Higher magnification shows a dominance of blast-like cells with only few maturing granulocytes in MYC/BCL-X_L_ mice (C and D) (primary magnification 60×).
(TIF)

Figure S4 **Immunohistochemical staining of sections of spleen and liver of Mock/MYC mice.** The section slides were stained with antibodies directed against the T-cell markers CD45R and CD3 and the myeloid marker myeloperoxidase (MPO) and analyzed by immunohistochemistry as described in Materials and Methods S1.
(TIF)

Figure S5 **Immunohistochemical staining of sections of spleen of Mock/Mock mice.** The analysis was performed as described in the legend to Figure S4.
(TIF)

Figure S6 **Immunohistochemical staining of sections of spleen and liver of MYC/BCL-X_L_ mice.** The analysis was performed as described in the legend to Figure S4.
(TIF)

Figure S7 **Immunohistochemical staining of sections of spleen and liver of MYC/BCL-2 mice.** The analysis was performed as described in the legend to Figure S4.
(TIF)

Figure S8 **Immunohistochemical staining of sections of spleen, liver and thymus of MYC/FLIP_L_ mice.** The analysis was performed as described in the legend to Figure S4.
(TIF)

Materials and Methods S1 **Immunohistochemistry.**
(DOCX)

Table S1 **Flow cytometric analysis of bone marrow, thymus, spleen and liver in MYC, BCL-X_L_,, BCL-2, FLIP_L_ or control virus recipient mice.**
(DOCX)

Table S2 **Differential count of nucleated cells in peripheral blood smears in MYC/BCL-XL and control mice.**
(DOCX)
