# Supplementary figures and images for: Inhibition of the Intrinsic but Not the Extrinsic Apoptosis Pathway Accelerates and Drives Myc-Driven Tumorigenesis Towards Acute Myeloid Leukemia
Source: PLoS One. 2012 Feb 29;7(2):e31366. doi: 10.1371/journal.pone.0031366 (PMC3290626; doi:10.1371/journal.pone.0031366)

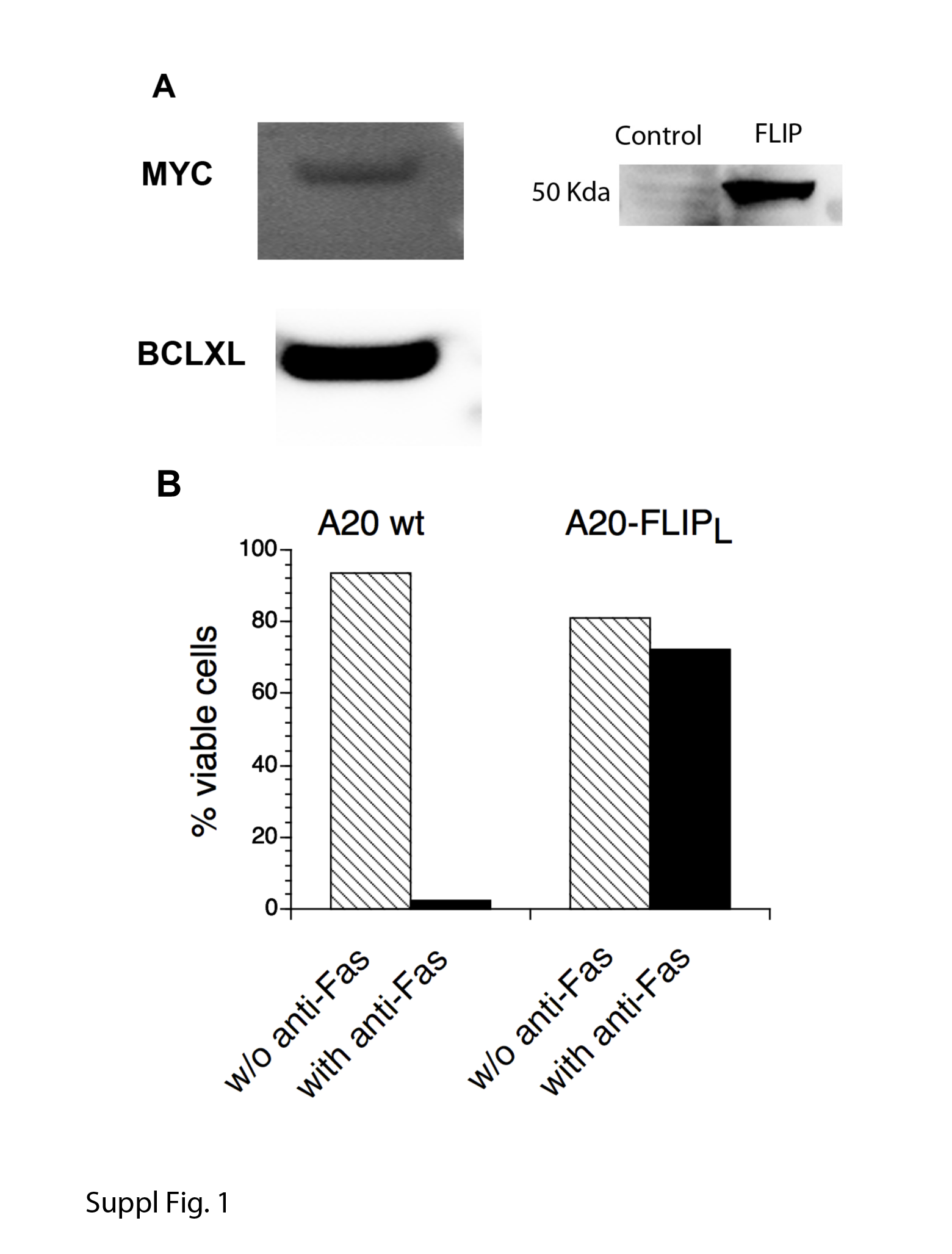

Supplement: Figure S1 — Expression and functionality of retroviral constructs. A. Analysis of proteins from MYC/BCL-XL tumor cells and Fas-sensitive A20 cells transduced with MSCV-hFLIPL-IRES-GFP retroviral particles. B. Fas-sensitive A20 cells were transduced with MSCV-hFLIPL-IRES-GFP retroviral particles. Transduced or wt A20 cells were thereafter incubated with or without agonistic anti-Fas mAb (0.25 µg/ml of Jo2) for 20 hours and thereafter analyzed by flow cytometry using propidium iodide (PI) to discriminate between live and dead cells. (TIF) [file pone.0031366.s001.tif]

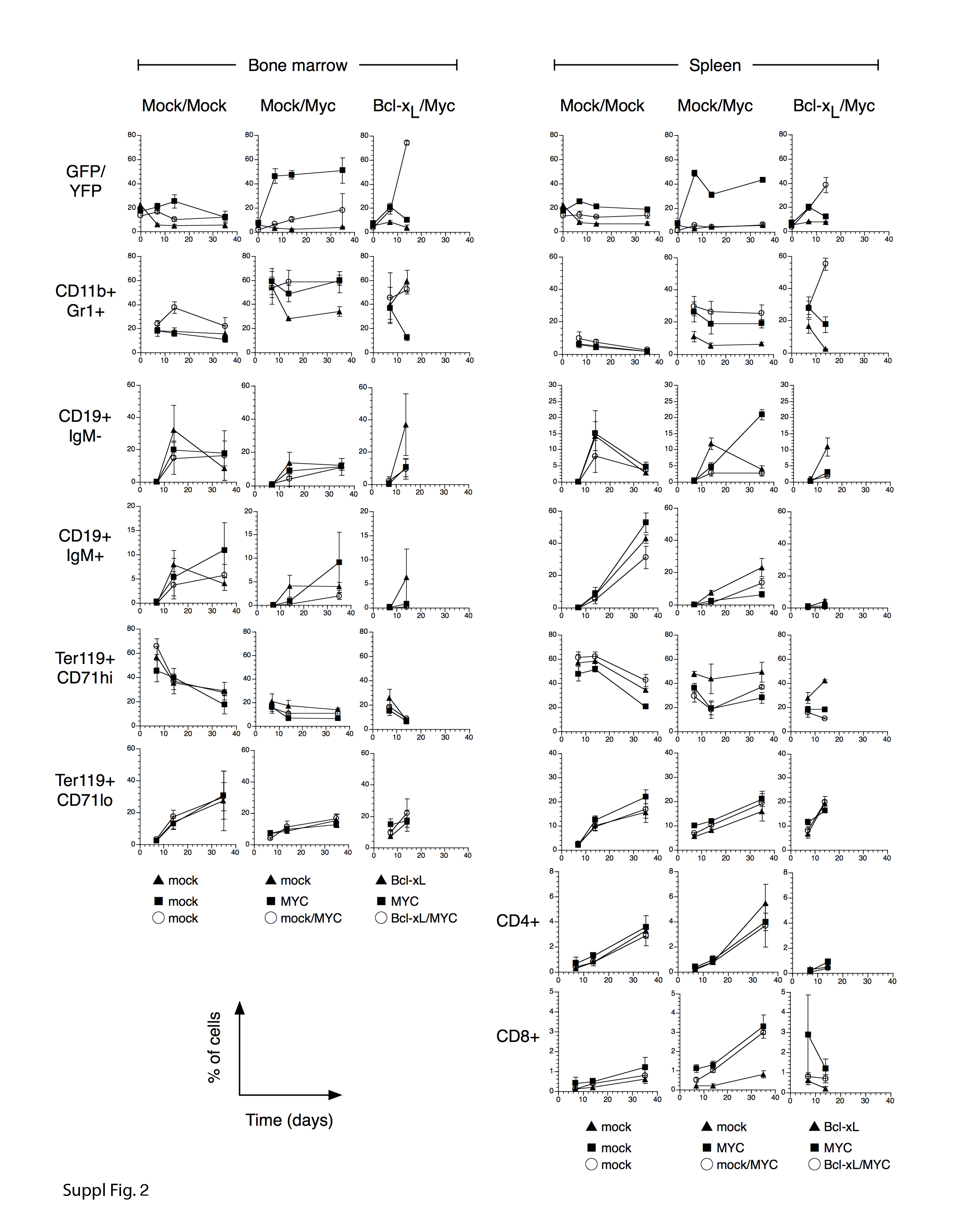

Supplement: Figure S2 — Early hematopoietic development of DBA/2 mice reconstituted with Mock-GFP/Mock-YFP, Mock-GFP/MYC-YFP or BCL-XL-GFP/Myc-YFP expressing HSCs. Phenotypic analysis of bone marrow (left) and spleen (right) at 7, 14 and 35 days after transplantation of Mock-GFP/Mock-YFP, Mock-GFP/MYC-YFP or BCL-XL-GFP/MYC-YFP expressing HSCs into DBA/2 mice was performed with FACS. The horizontal axis indicates days after transplantation and the vertical axis represents percentage of cells expressing the marker indicated to the left. Black triangles: GFP+YFP− cells, black squares; GFP−YFP+ cells and white circles; GFP+YFP+ cells. (TIF) [file pone.0031366.s002.tif]

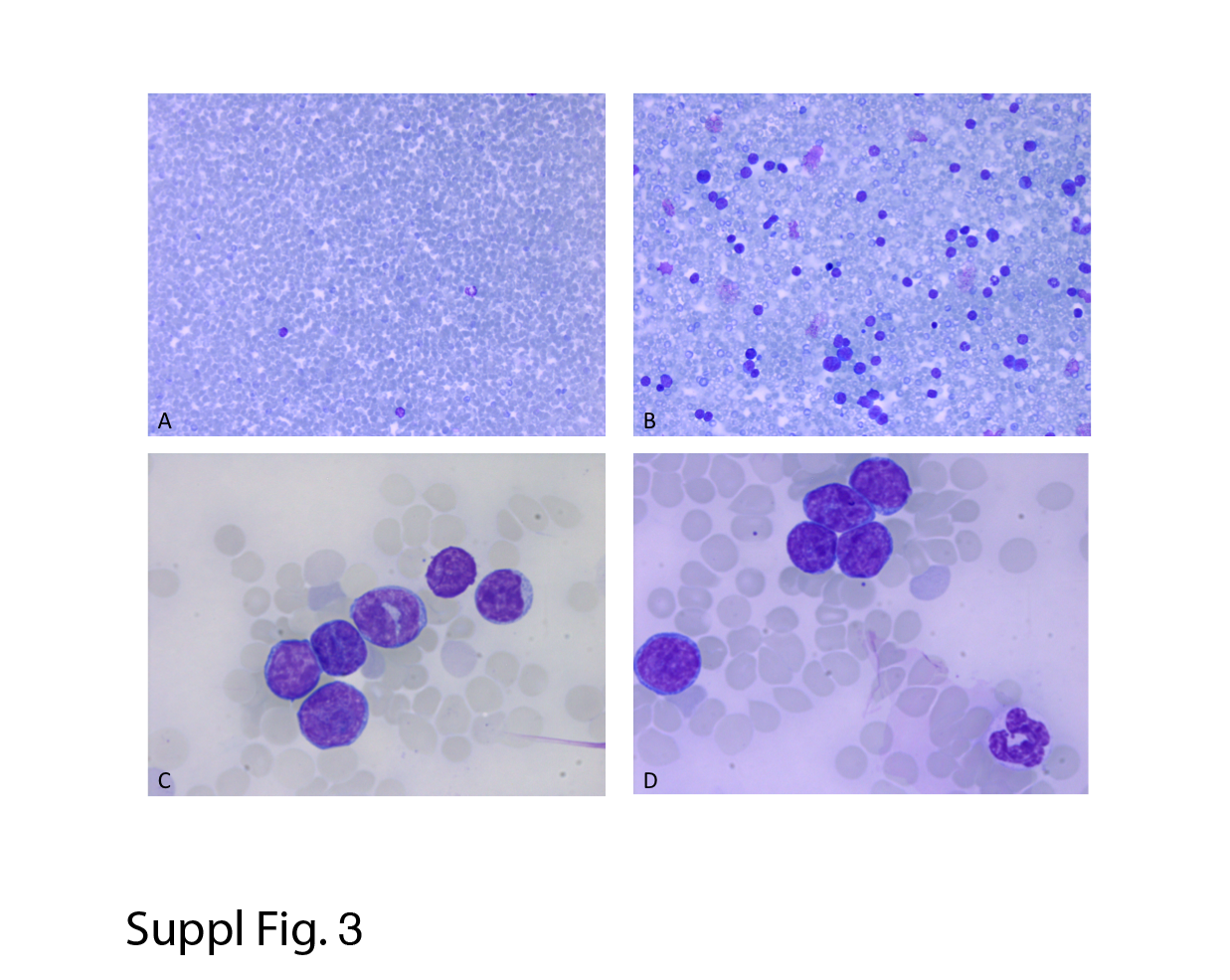

Supplement: Figure S3 — Appearance of leukemic blasts in the blood of MYC/BCL-XL recipient mice. Blood smears stained with May-Grunewald-Giemsa. Blood smear from a Mock/Mock mouse shows a normal picture with few leukocytes (A) as compared to the leukocytosis seen in MYC/BCL-XL mice (B) (primary magnification 20×). Higher magnification shows a dominance of blast-like cells with only few maturing granulocytes in MYC/BCL-XL mice (C and D) (primary magnification 60×). (TIF) [file pone.0031366.s003.tif]

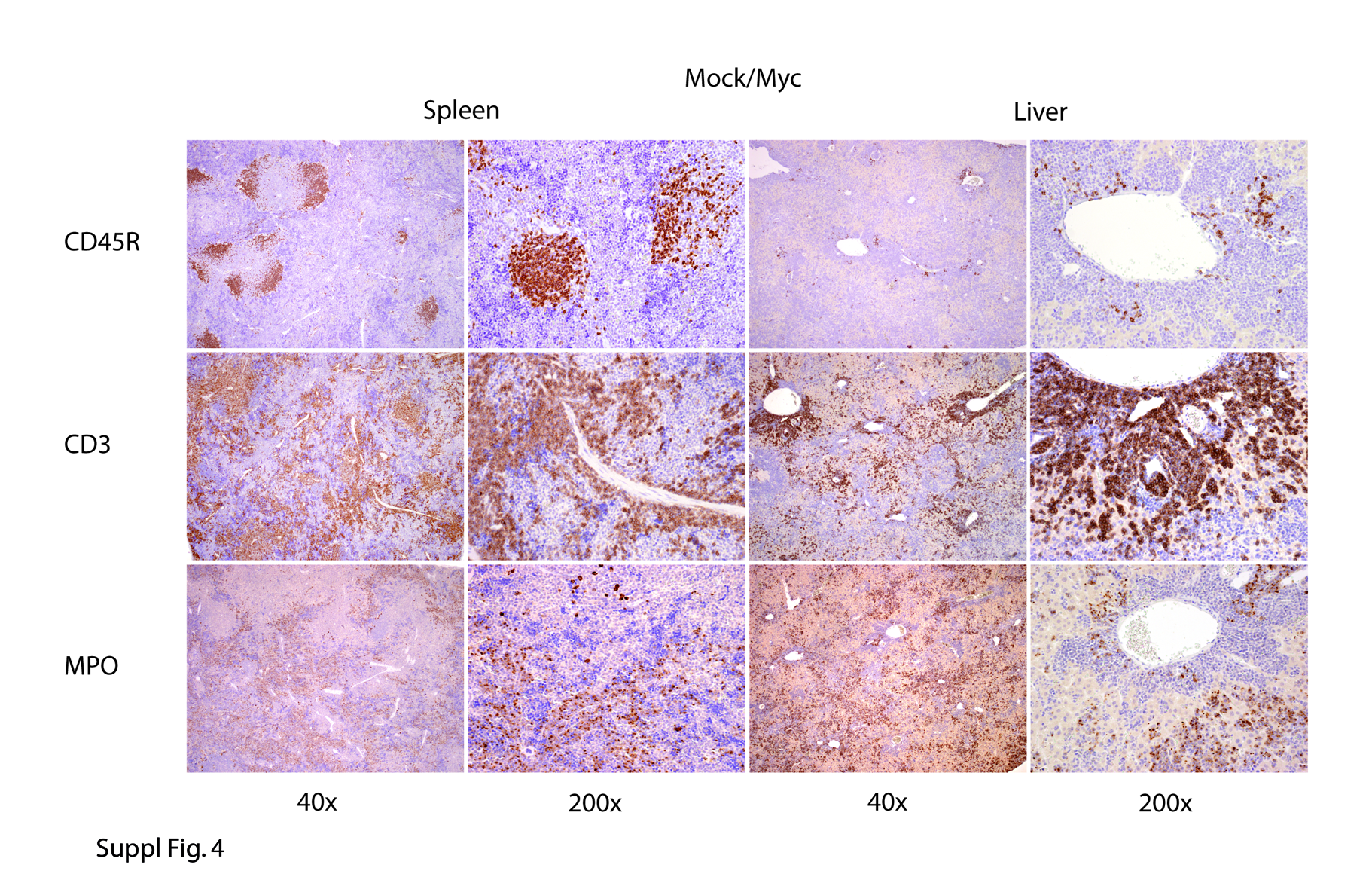

Supplement: Figure S4 — Immunohistochemical staining of sections of spleen and liver of Mock/MYC mice. The section slides were stained with antibodies directed against the T-cell markers CD45R and CD3 and the myeloid marker myeloperoxidase (MPO) and analyzed by immunohistochemistry as described in Materials and Methods S1. (TIF) [file pone.0031366.s004.tif]

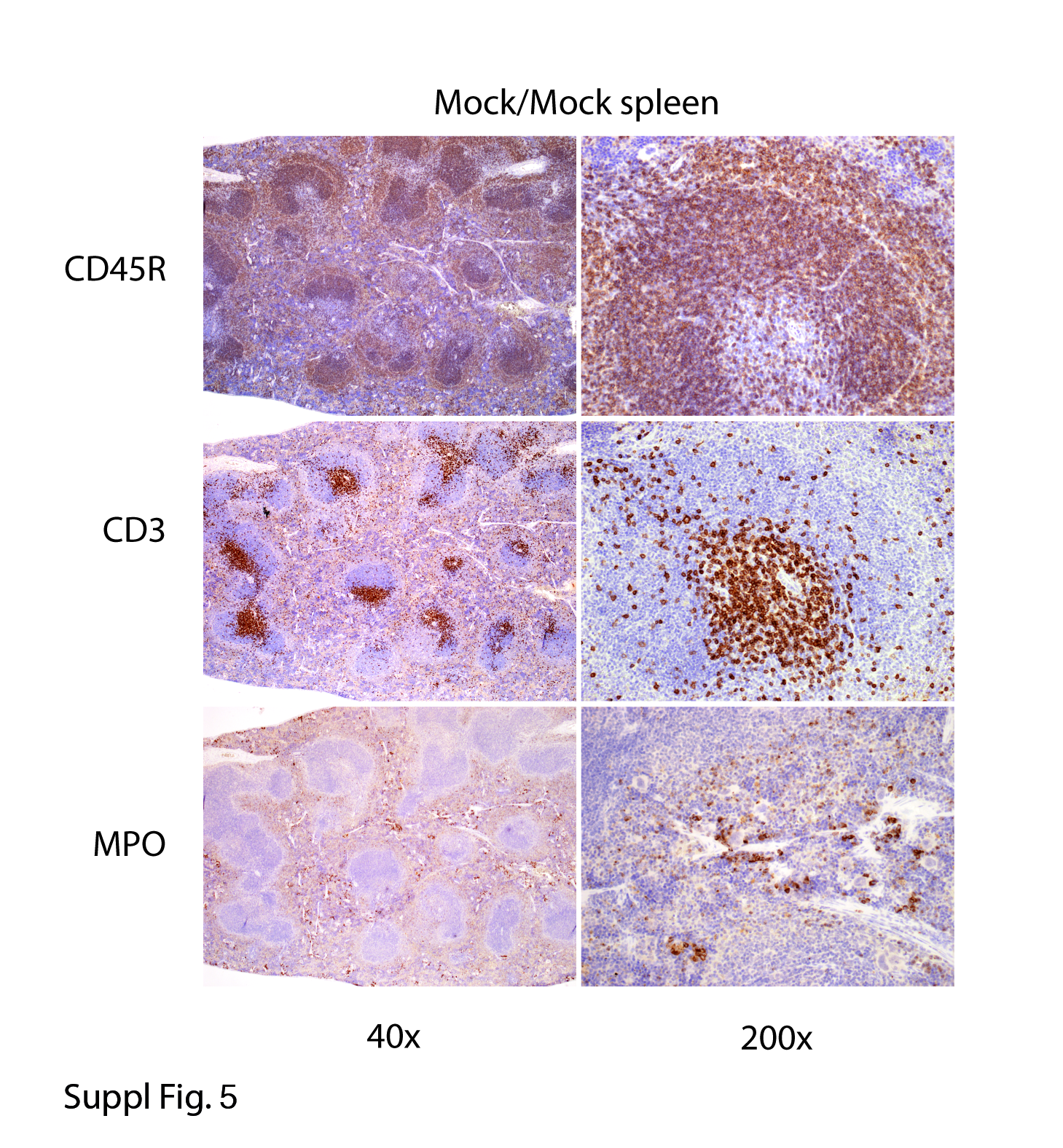

Supplement: Figure S5 — Immunohistochemical staining of sections of spleen of Mock/Mock mice. The analysis was performed as described in the legend to Figure S4. (TIF) [file pone.0031366.s005.tif]

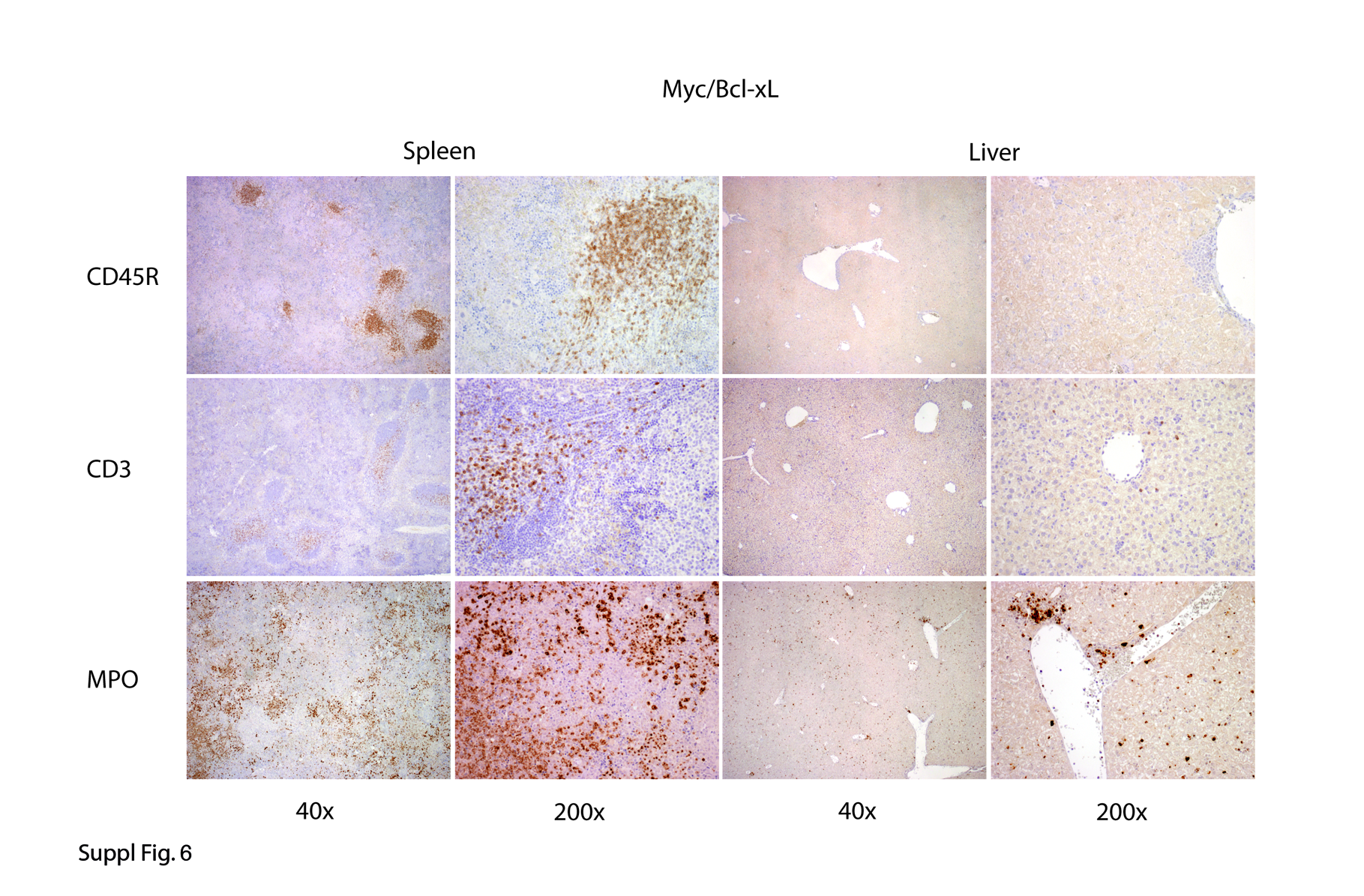

Supplement: Figure S6 — Immunohistochemical staining of sections of spleen and liver of MYC/BCL-XL mice. The analysis was performed as described in the legend to Figure S4. (TIF) [file pone.0031366.s006.tif]

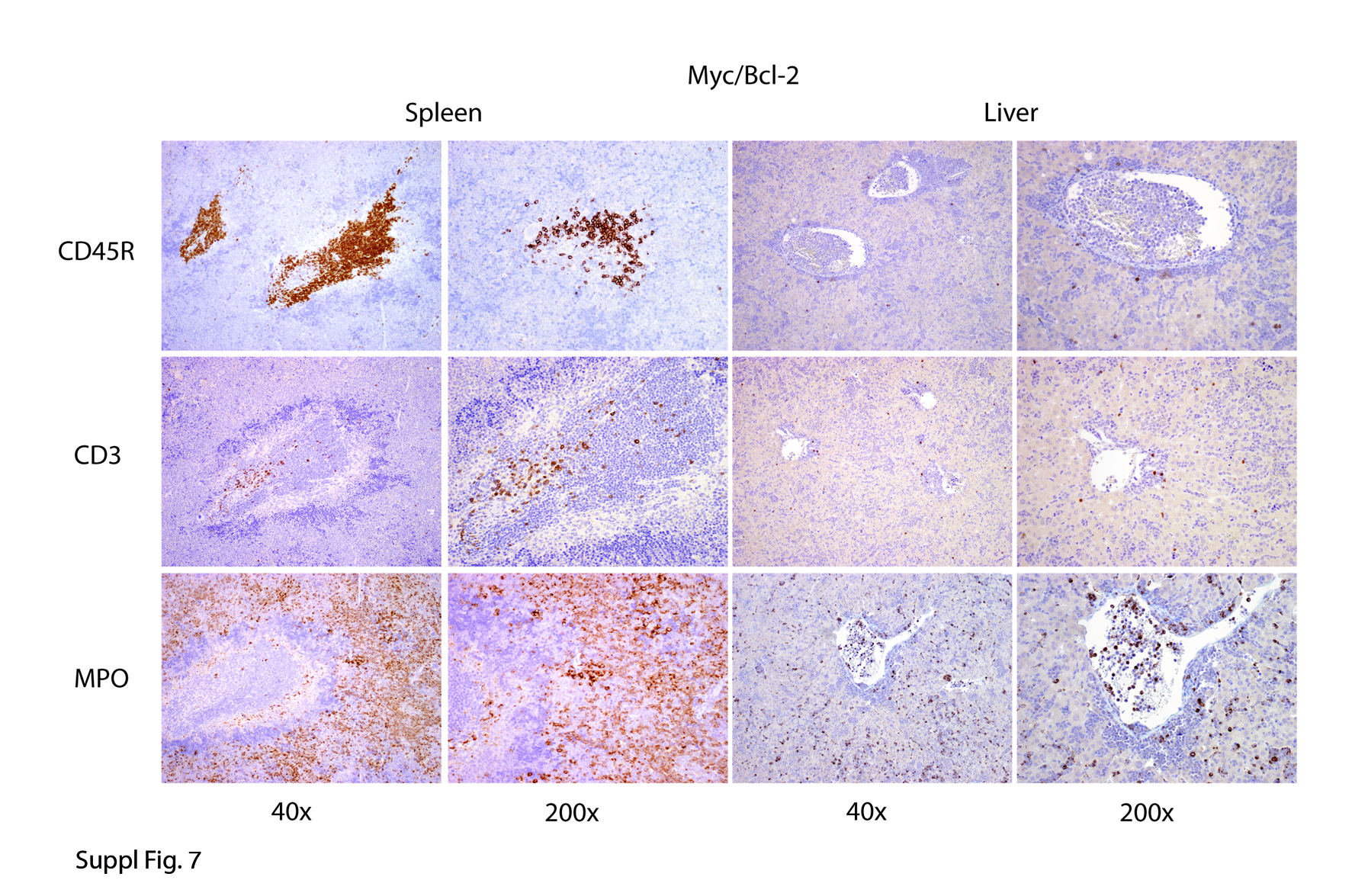

Supplement: Figure S7 — Immunohistochemical staining of sections of spleen and liver of MYC/BCL-2 mice. The analysis was performed as described in the legend to Figure S4. (TIF) [file pone.0031366.s007.tif]

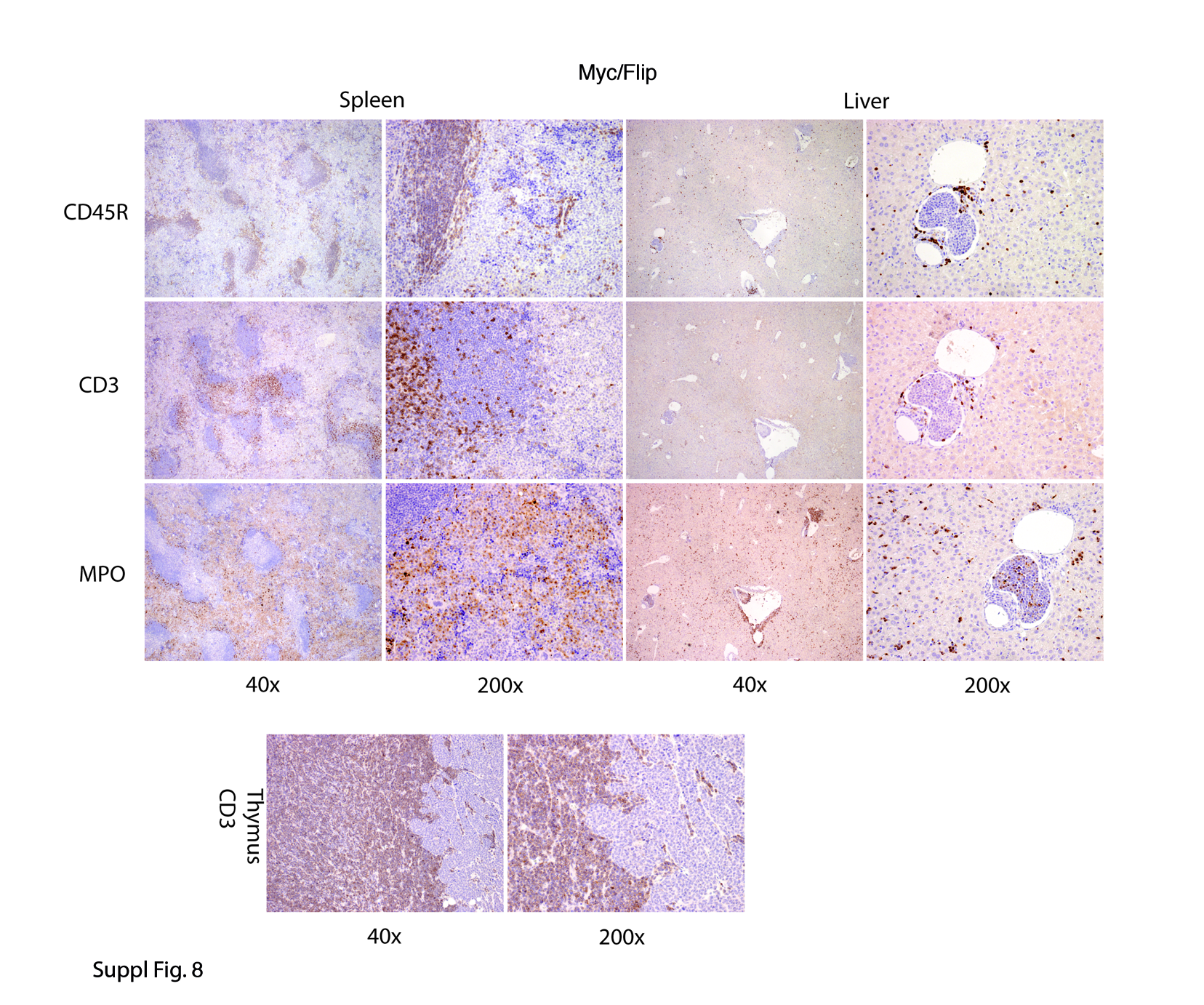

Supplement: Figure S8 — Immunohistochemical staining of sections of spleen, liver and thymus of MYC/FLIPL mice. The analysis was performed as described in the legend to Figure S4. (TIF) [file pone.0031366.s008.tif]
