# Supplementary material for: Inhibition of the Intrinsic but Not the Extrinsic Apoptosis Pathway Accelerates and Drives Myc-Driven Tumorigenesis Towards Acute Myeloid Leukemia
Source: PLoS One. 2012 Feb 29;7(2):e31366. doi: 10.1371/journal.pone.0031366 (PMC3290626; doi:10.1371/journal.pone.0031366)
Supplement: Materials and Methods S1 — Immunohistochemistry. (DOCX) [file pone.0031366.s009.docx]

**Materials and Methods S1**

**Immunohistochemistry**

Organs and tissues were fixed for 24 hours in neutral buffered formalin, dehydrated and processed to paraffin according to standard procedures. Paraffin sections were rehydrated and stained with hematoxylin/eosin or processed for immunohistochemistry. The following antibodies were used: Rabbit mAb against CD3 (Clone SP7, Labvision, Fremont, CA), rat monoclonal against CD45R/B220  (Clone RA3-6B2, BD Biosciences), rabbit polyclonal against myeloperoxidase (Dako, Glostrup, Denmark). Normal sera, biotinylated secondary antibodies and Streptavidin/HRP (Dako).

Briefly, sections were collected on plus-slides, dried and deparaffinized. Antigen retrieval was performed in a Retriever 2100 (PickCell Laboratories, The Netherlands). Endogenous peroxidase was quenched using methanol/hydrogen peroxide.  Before incubation with the primary antibodies sections were overlaid with 4 % normal serum of appropriate species.  Sections were incubated overnight at 4°C, and further processed with biotinylated secondary antibodies and finally exposed to strepavidin/HRP complex. Binding of primary mAb was visualized with DAB/hydrogen peroxide and the sections counterstained with hematoxylin. TNBS was used as a buffer in all incubations steps.
