# Supplementary material for: Inhibition of the Intrinsic but Not the Extrinsic Apoptosis Pathway Accelerates and Drives Myc-Driven Tumorigenesis Towards Acute Myeloid Leukemia
Source: PLoS One. 2012 Feb 29;7(2):e31366. doi: 10.1371/journal.pone.0031366 (PMC3290626; doi:10.1371/journal.pone.0031366)
Supplement: Table S1 — Flow cytometric analysis of bone marrow, thymus, spleen and liver in MYC, BCL-XL,, BCL-2, FLIPL or control virus recipient mice. (DOCX) [file pone.0031366.s010.docx]

**Table S1. Flow cytometric analysis of bone marrow, thymus, spleen and liver in MYC, BCL-X_L,_ BCL-2, FLIP_L_ and/or control virus recipient mice**.

**Table S1a. Flow cytometric analysis of bone marrow, thymus, spleen and liver in Mock-GFP/Mock-YFP recipient mice (DBA/2).**

|  | | | | | | | % expression of the indicated markers in YFP+GFP-, YFP-GFP+YFP+GFP+ expressing cells | | | | | | | | |
| --- | --- | --- | --- | --- | --- | --- | --- | --- | --- | --- | --- | --- | --- | --- | --- |
| Id | Type | Time of analysis (days) | Organ | Spleen weight/ Liver inf* | YFP+ GFP+ (%) | YFP+ GFP+ (%) | CD4+ CD8- (%) | CD8+ CD4- (%) | CD4+ CD8+ (%) | CD4- CD8- (%) | Gr-1+ CD11b+ (%) | Gr-1- CD11b+ (%) | Gr-1+ CD11b- (%) | CD19+ IgM+ (%) | CD19+ IgM- (%) |
| 118 | Mock/Mock | 49 | BM |  | 54.6# |  | <1 | <1 | <1 | n.a. | 25.6 | 5.1 | 1 | 7.6 | 14.8 |
| = | = | = | THY |  | 49.6# |  | 4.1 | <1 | 83.9 | 7.0 | n.a. | n.a. | n.a. | n.a. | n.a. |
| = | = | = | SPL | 148 | 36# |  | 5 | 1 | <1 | n.a. | 4 | 3 | <1 | 28 | 2 |
| = | = | = | LIV | 2.7 | 49.0# |  | 1.5 | <1 | <1 | n.a. | 1.4 | 1.2 | <1 | 2.2 | <1 |
| 119 | = | = | BM |  | 34.8# |  | <1 | <1 | <1 | n.a. | 29.4 | 6.3 | 1.2 | 3.2 | 5.0 |
| = | = | = | THY |  | 17.0# |  | 6.4 | 1.2 | 65.4 | 22.9 | n.a. | n.a. | n.a. | n.a. | n.a. |
| = | = | = | SPL | 144 | 30# |  | 6 | 1 | <1 | n.a. | 4 | 4 | <1 | 33 | 2 |
| = | = | = | LIV | 3.3 | 45.6 |  | <1 | <1 | <1 | n.a. | <1 | <1 | <1 | 2.5 | <1 |
| 120 | = | = | BM |  | 39.2# |  | <1 | <1 | <1 | n.a. | 25.1 | 5.0 | <1 | 5.0 | 8.8 |
| = | = | = | THY |  | 24.6# |  | 5.6 | 1.1 | 70.1 | 17.9 | n.a. | n.a. | n.a. | n.a. | n.a. |
| = | = | = | SPL | 178 | 31# |  | 7 | 2 | <1 | n.a. | 3 | 4 | <1 | 33 | 2 |
| = | = | = | LIV | 3.4 | 45.5# |  | 1.4 | <1 | <1 | n.a. | <1 | <1 | <1 | 2.7 | <1 |

**Table S1b: Flow cytometric analysis of bone marrow, thymus, spleen and liver in Mock-GFP/Myc-YFP recipient mice (DBA/2).**

|  | | | | | | | % expression of the indicated markers in YFP+GFP- and YFP+GFP+ cell populations as indicated | | | | | | | | |
| --- | --- | --- | --- | --- | --- | --- | --- | --- | --- | --- | --- | --- | --- | --- | --- |
| Id | Type | Time of analysis (days) | Organ | Spleen weight/ Liver inf* | YFP+ GFP- (%) | YFP+ GFP+ (%) | CD4+ CD8- (%) | CD8+ CD4- (%) | CD4+ CD8+ (%) | CD4- CD8- (%) | Gr-1+ CD11b+ (%) | Gr-1- CD11b+ (%) | Gr-1+ CD11b- (%) | CD19+ IgM+ (%) | CD19+ IgM- (%) |
| 173 | Mock/Myc | 49 | BM |  | 52 |  | **16** | 4 | 1.5 | n.a. | 32 | 6 | 5 | 3 | <1 |
| = | = | = | THY |  | 69.8 |  | **49.5** | <1 | 40.2 | 9.1 | <1 | <1 | <1 | n.a. | n.a. |
| = | = | = | SPL | 900 | 52 |  | **16** | 4 | 1.5 | n.a. | **32** | 6 | *5* | 3 | <1 |
| = | = | = | LIV | 8.4 | 46.1 |  | **13.1** | *9.1* | *2.1* | n.a. | **38.8** | **13.4** | *3.6* | 1.2 | <1 |
| 174 | = | = | BM |  | 41.5 |  | 1.1 | 1.3 | <1 | n.a. | **58** | 8.3 | 1.9 | 2.0 | 4.2 |
| = | = | = | THY |  | 94.5 |  | 1.8 | 6.1 | 91.5 | <1 | <1 | <1 | <1 | n.a. | n.a. |
| = | = | = | SPL | 1042 | 26 |  | 2 | 2 | <1 | n.a. | **25** | 5 | 2 | 15 | 1 |
| = | = | = | LIV | 21.1 | 30.2 |  | 3.3 | 3.6 | 0.4 | n.a. | **39.1** | **6.1** | *3.2* | 5.8 | <1 |
| 175 | = | = | BM |  | 43.2 |  | 1.1 | 1.6 | **3.8** | n.a. | 20.2 | 5.2 | 1.4 | <1 | <1 |
| = | = | = | = |  |  | 20.9 | <1 | 1 | 2.5 | n.a. | 21.3 | 5.8 | <1 | <1 | <1 |
| = | = | = | THY |  | 39.7 |  | **43.6** | 6.3 | 38.9 | 11.2 | <1 | <1 | <1 | n.a. | n.a. |
| = | = | = | SPL | 744 | 59.7 |  | 7.2 | 8 | **47.4** | n.a. | 2.9 | 2.7 | <1 | 3.2 | <1 |
| = | = | = | = |  |  | 8.5 | <1 | *10.2* | **32.1** | n.a. | *12.3* | 6.5 | <1 | 8.3 | 2.4 |
| = | = | = | LIV | 13.5 | 60.8 |  | *7.4* | *7.3* | **47.5** | n.a. | **8.7** | *3.5* | 1.1 | 4 | <1 |
| = | = | = | = |  |  | 8.4 | <1 | 2,5 | **32.2** | n.a. | **23.5** | *7.2* | <1 | 4 | <1 |
| 176 | = | 54 | BM |  | 66 |  | <1 | <1 | <1 | n.a. | **49.6** | 3.5 | <1 | <1 | <1 |
| = | = | = | = |  |  | 12.6 | <1 | <1 | <1 | n.a. | **51.8** | 11 | <1 | 1.1 | <1 |
| = | = | = | THY |  | 14 |  | *13.8* | 4.6 | 70 | 11.5 | **5** | <1 | <1 | n.a. | n.a. |
| = | = | = | SPL | 446 | 35 |  | 6 | 2 | **17** | n.a. | **23** | 5 | <1 | 16 | 3 |
| = | = | = | = |  |  | 27 | <1 | 1 | 1 | n.a. | **12** | **66** | <1 | 3.5 | <1 |
| = | = | = | LIV | 6 | 32.5 |  | *2.9* | *7.9* | **6** | n.a. | **42.4** | *8.4* | 1.4 | 1.9 | <1 |
| = | = | = | = |  |  | 32.1 | <1 | 1.1 | <1 | n.a. | **14.6** | **70.5** | <1 | <1 | <1 |
|  | | | | | | | % expression of the indicated markers in YFP+GFP- and YFP+GFP+ cell populations as indicated | | | | | | | | |
| Id | Type | Time of analysis (days) | Organ | Spleen weight/ Liver inf* | YFP+ GFP- (%) | YFP+ GFP+ (%) | CD4+ CD8- (%) | CD8+ CD4- (%) | CD4+ CD8+ (%) | CD4- CD8- (%) | Gr-1+ CD11b+ (%) | Gr-1- CD11b+ (%) | Gr-1+ CD11b- (%) | CD19+ IgM+ (%) | CD19+ IgM- (%) |
| 178 | Mock/Myc | 58 | BM |  | 40.6 |  | 4.9 | 2.9 | <1 | n.a. | 42.3 | 22.3 | 2.2 | n.a. | n.a. |
| = | = | = | THY |  | 78.5 |  | 15.3 | 1.1 | 80 | 3.6 | <1 | 58.8lo | <1 | n.a. | n.a. |
| = | = | = | SPL | 1446 | 25.5 |  | 5.6 | 1.8 | <1 | n.a. | 16.6 | 6.9 | 3.2 | 2.3 | <1 |
| = | = | = | = |  |  | 7.3 | 2.6 | 1 | <1 | n.a. | 83 | 6.8 | 4.6 | <1 | <1 |
| = | = | = | LIV | 6.7 | 36.6 |  | 5.5 | 5.0 | 2.4 | n.a. | 37.6 | 11.2 | 2.7 | n.a. | n.a. |
| = | = | = | = |  |  | 19.1 | 2.3 | 2.6 | <1 | n.a. | 81.7 | 6.7 | 2.2 | n.a. | n.a. |
| 179 | = | = | BM |  | 70 |  | 36.3 | 1.5 | 30.2 | n.a. | 14.6 | 3.1 | 7 | n.a. | n.a. |
| = | = | = | = |  |  | 10.5 | 3.1 | 4 | 12.7 | n.a. | 59.7 | 20.7 | 1.3 | n.a. | n.a. |
| = | = | = | THY |  | n.a. | n.a. | n.a. | n.a. | n.a. | n.a. | n.a. | n.a. | n.a. | n.a. | n.a. |
| = | = | = | SPL | 854 | 45 |  | 23 | 2 | 27 | n.a. | 10 | 3 | 3 | 7 | <1 |
| = | = | = | LIV | 10.6 | 64.3 |  | 5.8 | <1 | 64.9 | n.a. | 20.7 | 3.2 | 2.6 | n.a. | n.a. |
| 180 | = | 61 | BM |  | 53.2 |  | 11.6 | <1 | <1 | n.a. | 20.8 | 3.1 | 1.6 | 1 | 1.6 |
| = | = | = | = |  |  | 24.2 | <1 | <1 | <1 | n.a. | 22.6 | 9.3 | <1 | <1 | <1 |
| = | = | = | THY |  | 92.5 |  | 96.3 | <1 | 1.4 | 2.3 | <1 | <1 | <1 | n.a. | n.a. |
| = | = | = | SPL | 443 | 49.9 |  | 28.8 | 3.2 | <1 | n.a. | 1.5 | 1.1 | 5.1 | 5.3 | 9.1 |
| = | = | = | = |  |  | 9.2 | 3 | 8.2 | 1.4 | n.a. | 6.4 | 1.9 | 16.6 | 4.2 | 10.8 |
| = | = | = | LIV | 3.8 | 59.7 |  | 13.8 | 1.6 | <1 | n.a. | 10.6 | 7.2 | 6.4 | 4.9 | 1.4 |
| = | = | = | = |  |  | 13.3 | <1 | <1 | <1 | n.a. | 33.9 | 10.3 | 9.3 | 3.4 | <1 |
| 181 | = | 63 | BM |  | 45 |  | 1.4 | 11.1 | <1 | n.a. | 36.2 | 13.9 | 15.4 | 1 | <1 |

|  | | | | | | | % expression of the indicated markers in YFP+GFP- and YFP+GFP+ cell populations as indicated | | | | | | | | |
| --- | --- | --- | --- | --- | --- | --- | --- | --- | --- | --- | --- | --- | --- | --- | --- |
| Id | Type | Time of analysis (days) | Organ | Spleen weight/ Liver inf* | YFP+ GFP- (%) | YFP+ GFP+ (%) | CD4+ CD8- (%) | CD8+ CD4- (%) | CD4+ CD8+ (%) | CD4- CD8- (%) | Gr-1+ CD11b+ (%) | Gr-1- CD11b+ (%) | Gr-1+ CD11b-(%) | CD19+ IgM+ (%) | CD19+ IgM- (%) |
| 178 | Mock/Myc | 58 | BM |  | 40.6 |  | 4.9 | 2.9 | <1 | n.a. | *42.3* | **22.3** | 2.2 | n.a. | n.a. |
| = | = | = | THY |  | 78.5 |  | *15.3* | 1.1 | 80 | 3.6 | <1 | *58.8^lo^* | <1 | n.a. | n.a. |
| = | = | = | SPL | 1446 | 25.5 |  | 5.6 | 1.8 | <1 | n.a. | **16.6** | *6.9* | 3.2 | 2.3 | <1 |
| = | = | = | = |  |  | 7.3 | 2.6 | 1 | <1 | n.a. | **83** | *6.8* | 4.6 | <1 | <1 |
| = | = | = | LIV | 6.7 | 36.6 |  | *5.5* | *5.0* | 2.4 | n.a. | **37.6** | **11.2** | 2.7 | n.a. | n.a. |
| = | = | = | = |  |  | 19.1 | 2.3 | 2.6 | <1 | n.a. | **81.7** | 6.7 | 2.2 | n.a. | n.a. |
| 179 | = | = | BM |  | 70 |  | **36.3** | 1.5 | **30.2** | n.a. | 14.6 | 3.1 | *7* | n.a. | n.a. |
| = | = | = | = |  |  | 10.5 | 3.1 | 4 | **12.7** | n.a. | **59.7** | **20.7** | 1.3 | n.a. | n.a. |
| = | = | = | THY |  | n.a. | n.a. | n.a. | n.a. | n.a. | n.a. | n.a. | n.a. | n.a. | n.a. | n.a. |
| = | = | = | SPL | 854 | 45 |  | **23** | 2 | **27** | n.a. | *10* | 3 | 3 | 7 | <1 |
| = | = | = | LIV | 10.6 | 64.3 |  | *5.8* | <1 | **64.9** | n.a. | **20.7** | 3.2 | 2.6 | n.a. | n.a. |
| 180 | = | 61 | BM |  | 53.2 |  | **11.6** | <1 | <1 | n.a. | 20.8 | 3.1 | 1.6 | 1 | 1.6 |
| = | = | = | = |  |  | 24.2 | <1 | <1 | <1 | n.a. | 22.6 | 9.3 | <1 | <1 | <1 |
| = | = | = | THY |  | 92.5 |  | **96.3** | <1 | 1.4 | 2.3 | <1 | <1 | <1 | n.a. | n.a. |
| = | = | = | SPL | 443 | 49.9 |  | **28.8** | 3.2 | <1 | n.a. | 1.5 | 1.1 | 5.1 | 5.3 | 9.1 |
| = | = | = | = |  |  | 9.2 | 3 | *8.2* | 1.4 | n.a. | 6.4 | 1.9 | 16.6 | 4.2 | 10.8 |
| = | = | = | LIV | 3.8 | 59.7 |  | **13.8** | 1.6 | <1 | n.a. | **10.6** | *7.2* | 6.4 | 4.9 | 1.4 |
| = | = | = | = |  |  | 13.3 | <1 | <1 | <1 | n.a. | **33.9** | **10.3** | 9.3 | 3.4 | <1 |

|  | | | | | | | % expression of the indicated markers in YFP+GFP- and YFP+GFP+ cell populations as indicated | | | | | | | | |
| --- | --- | --- | --- | --- | --- | --- | --- | --- | --- | --- | --- | --- | --- | --- | --- |
| Id | Type | Time of analysis (days) | Organ | Spleen weight/ Liver inf* | YFP+ GFP- (%) | YFP+ GFP+ (%) | CD4+ CD8- (%) | CD8+CD4- (%) | CD4+ CD8+ (%) | CD4- CD8- (%) | Gr-1+ CD11b+ (%) | Gr-1- CD11b+ (%) | Gr-1+ CD11b-(%) | CD19+ IgM+ (%) | CD19+ IgM- (%) |
| 181 | Mock/Myc | 63 | BM |  | 45 |  | 1.4 | **11.1** | <1 | n.a. | *36.2* | **13.9** | **15.4** | 1 | <1 |
| = | = | = | = |  |  | 30.9 | <1 | 2.1 | <1 | n.a. | *33.2* | **18.7** | 2.2 | 1 | 2 |
| = | = | = | THY |  |  | 85.1 | **87.7** | <1 | 10.2 | 2.0 | <1 | <1 | <1 | n.a. | n.a. |
| = | = | = | SPL | 716 | 50 |  | **22.7** | **24.5** | 1.2 | n.a. | **18.4** | 7.7 | **16.4** | 1.8 | <1 |
| = | = | = | = |  |  | 5 | 6.7 | 8.5 | <1 | n.a. | 8.1 | 9.1 | <1 | 10.3 | 3.7 |
| = | = | = | LIV | 5.6 | 42.6 |  | **20** | 2.6 | <1 | n.a. | **49.5** | 2.5 | **30.9** | n.a. | n.a. |
| 182 | = | 65 | BM |  | 71.4 |  | **24.8** | 2 | **6** | n.a. | 23.1 | 18.8 | <1 | <1 | <1 |
| = | = | = | = |  |  | 15.2 | <1 | 5.4 | **12.1** | n.a. | 5.5 | 2.9 | <1 | <1 | <1 |
| = | = | = | THY |  | 94.1 |  | **93.8** | <1 | 4.1 | 2 | <1 | <1 | <1 | n.a. | n.a. |
| = | = | = | SPL | 548 | 46 |  | 8 | 4 | <1 | n.a. | 9 | 6 | 2 | 16 | 4 |
| = | = | = | = |  |  | 13 | **28** | 1 | <1 | n.a. | **41** | **10** | 6 | 7 | 1 |
| = | = | = | LIV | 7 | 31.7 |  | *6.3* | 2.6 | <1 | n.a. | **17.9** | **9.4** | *4.8* | n.a. | n.a. |
| 183 | = | = | BM |  | 73.4 |  | **15.2** | 1.2 | 1.4 | n.a. | 23.6 | 8.7 | 1.8 | n.a. | n.a. |
| = | = | = | = |  |  | 10.5 | <1 | 1.4 | 1.9 | n.a. | 30.7 | 13.9 | <1 | n.a. | n.a. |
| = | = | = | THY | 91.3 |  |  | **44.6** | 2.7 | 16.8 | 35.8 | <1 | <1 | <1 | n.a. | n.a. |
| = | = | = | SPL | 1252 | 75 |  | **16** | 2 | <1 | n.a. | 2 | 1 | 1 | 3 | 1 |
| = | = | = | = |  |  | 9.2 | 3 | 8.2 | 1.4 | n.a. | 6.4 | 1.9 | **16.6** | 4.2 | 10.8 |
| = | = | = | LIV | 11.7 | 71.3 |  | **12.6** | 1.4 | <1 | n.a. | 3.3 | 1.5 | 1.4 | n.a. | n.a. |
| 184 | = | = | BM |  | 84.1 |  | 1.2 | **40.4** | *2.3* | n.a. | 24.8 | 7.4 | **35.2** | n.a. | n.a. |
| = | = | = | THY |  | 82.6 |  | 2.2 | *15.3* | 80.6 | 1.9 | n.a. | n.a. | n.a. | n.a. | n.a. |
| = | = | = | SPL | 764 | 65 |  | 13 | **45** | **13** | n.a. | 2 | 4 | 9 | 5 | 1 |
| = | = | = | LIV | 8.6 | 63.9 |  | 1.3 | **78.2** | 1.1 | n.a. | 4.2 | 0.6 | **77.9** | n.a. | n.a. |

**Table S1c: Flow cytometric analysis of bone marrow, thymus, spleen and liver in FLIP_L_-GFP/Myc-YFP recipient mice (DBA/2).**

|  | | | | | | | % expression of the indicated markers in YFP+GFP- and YFP+GFP+ cell populations as indicated | | | | | | | | |
| --- | --- | --- | --- | --- | --- | --- | --- | --- | --- | --- | --- | --- | --- | --- | --- |
| Id | Type | Time of analysis (days) | Organ | Spleen weight/ Liver inf* | YFP+ GFP- (%) | YFP+ GFP+ (%) | CD4+ CD8- (%) | CD8+ CD4- (%) | CD4+ CD8+ (%) | CD4- CD8- (%) | Gr-1+ CD11b+ (%) | Gr-1- CD11b+ (%) | Gr-1+ CD11b-(%) | CD19+ IgM+ (%) | CD19+ IgM-(%) |
| 96 | FLIP/Myc | 48 | BM |  |  | 75 | n.a. | n.a. | n.a. | n.a. | <1 | <1 | <1 | <1 | <1 |
| = | = | = | THY |  |  | 72 | **91.1** | <1 | <1 | 8.9 | n.a. | n.a. | n.a. | n.a. | n.a. |
| = | = | = | SPL | 694 |  | 45 | **77** | <1 | <1 | <1 | <1 | <1 | <1 | <1 | <1 |
| = | = | = | LIV | 15 |  | 81 | **90.9** | <1 | <1 | n.a. | <1 | <1 | <1 | <1 | <1 |
| 97 | = | 49 | BM |  |  | 66.5 | n.a. | n.a. | n.a. | n.a. | 10.1 | 1.2 | 6.6 | <1 | <1 |
| = | = | = | THY |  | 26.7 |  | **42.1** | 1.4 | 53.5 | 2.9 | n.a. | n.a. | n.a. | n.a. | n.a. |
| = | = | = | SPL | 502 |  | 53 | **22** | <1 | <1 | n.a. | 1 | <1 | <1 | <1 | <1 |
| = | = | = | LIV | 7.1 | 16 |  | **28.7** | *7.9* | 1.0 | n.a. | **28.4** | **17.7** | **6.3** | <1 | <1 |
| = | = | = | = |  |  | 49.7 | **25.8** | <1 | <1 | n.a. | 4 | 1.6 | 1.9 | <1 | <1 |
| 98 | = | = | BM |  | 90.5 |  | n.a. | n.a. | n.a. | n.a. | 7.5 | 8.4 | <1 | <1 | <1 |
| = | = | = | THY |  | 47.4 |  | 7.9 | <1 | 86.5 | 4.7 | n.a. | n.a. | n.a. | n.a. | n.a. |
| = | = | = | SPL | 496 | 46 |  | 9 | 7 | 9 | n.a. | *13* | **35** | 2 | 6 | 1 |
| = | = | = | LIV | 9 | 50.2 |  | *6.9* | *6.4* | **24.1** | n.a. | **32.7** | **14** | 3.7 | 1.9 | 1.1 |
| 99 | = | = | BM |  | 49.6 |  | n.a. | n.a. | n.a. | n.a. | **62.9** | **13.5** | 2.5 | <1 | 1.9 |
| = | = | = | THY |  | 24.1 |  | 4 | 9.5 | 38.3 | 48.2 | n.a. | n.a. | n.a. | n.a. | n.a. |
| = | = | = | = |  |  | 37 | <1 | 8.4 | 55.2 | 35.5 | n.a. | n.a. | n.a. | n.a. | n.a. |
| = | = | = | SPL | 344 | 26.1 |  | 4 | 2.7 | <1 | n.a. | *9.9* | **14.9** | <1 | 14 | 3 |
| = | = | = | = |  |  | 3.7 | <1 | <1 | <1 | n.a. | *12.5* | **62.6** | <1 | 3 | <1 |
| = | = | = | LIV | 3.8 | 16.6 |  | *4.7* | *6.2* | *2.8* | n.a. | **55.1** | **18.7** | 2.7 | 3.7 | <1 |

|  | | | | | | | % expression of the indicated markers in YFP+GFP- and YFP+GFP+ cell populations as indicated | | | | | | | | |
| --- | --- | --- | --- | --- | --- | --- | --- | --- | --- | --- | --- | --- | --- | --- | --- |
| Id | Type | Time of analysis (days) | Organ | Spleen weight/ Liver inf* | YFP+ GFP (%) | YFP+ GFP+ (%) | CD4+ CD8- (%) | CD8+ CD4- (%) | CD4+ CD8+ (%) | CD4- CD8- (%) | Gr-1+ CD11b+ (%) | Gr-1- CD11b+ (%) | Gr-1+ CD11b-(%) | CD19+ IgM+ (%) | CD19+ IgM- (%) |
| 101 | FLIP/Myc | 49 | BM |  | 38.8 |  | n.a. | n.a. | n.a. | n.a. | **49.7** | 8.9 | <1 | 2.2 | 7.8 |
| = | = | = | = |  |  | 15.5 | n.a. | n.a. | n.a. | n.a. | **73.8** | 8.9 | <1 | 1.8 | 4.4 |
| = | = | = | THY |  | 22.9 |  | **18.5** | 1.9 | 76.9 | 2.7 | n.a. | n.a. | n.a. | n.a. | n.a. |
| = | = | = | SPL | 300 | 44 |  | **39** | 1 | 2 | n.a. | 4 | 4 | <1 | 14 | 4 |
| = | = | = | LIV | 3.1 | 19.2 |  | **28.8** | *3.4* | *1.6* | n.a. | **22.4** | **10.9** | **28.4** | n.a. | n.a. |
| 102 | = | 51 | BM |  | 53.9 |  | n.a. | n.a. | n.a. | n.a. | **49.6** | 10.2 | 1.2 | <1 | 3.3 |
| = | = | = | THY |  | 51.3 |  | **24.4** | <1 | 73.6 | 1.2 | n.a. | n.a. | n.a. | n.a. | n.a. |
| = | = | = | SPL | 514 | 45.7 |  | 4.1 | 1.7 | 1.2 | n.a. | **36.2** | **34.1** | <1 | 2.6 | <1 |
| = | = | = | LIV | 7.2 | 16 |  | **28.7** | *7.9* | 1.0 | n.a. | **28.4** | **17.7** | *6.3* | <1 | <1 |
| 103 | = | 56 | BM |  |  | 53 | **37.8** | <1 | <1 | n.a. | 19.7 | 4.6 | 3.6 | <1 | <1 |
| = | = | = | THY |  |  | 36.1 | **97.4** | <1 | 2 | <1 | <1 | <1 | **22.1** | n.a. | n.a. |
| = | = | = | SPL | 828 |  | 33 | **55** | <1 | <1 | n.a. | **36** | 2 | 3 | 2 | <1 |
| = | = | = | LIV | 7.6 |  | 31.6 | **16.8** | <1 | <1 | n.a. | **62** | *5.5* | *4.6* | <1 | <1 |
| 104 | = | = | BM |  |  | 63.9 | 1.7 | <1 | <1 | n.a. | n.a. | n.a. | n.a. | <1 | <1 |
| = | = | = | THY |  |  | 67.5 | **25.4** | <1 | 4.4 | 70.1 | <1 | 2.2 | 2.2 | n.a. | n.a. |
| = | = | = | SPL | 850 |  | 35 | **14** | 1 | <1 | n.a. | 4 | 3 | 7 | 1 | <1 |
| = | = | = | LIV | 13.5 |  | 38.5 | **18.6** | 2.1 | <1 | n.a. | **9** | **7.8** | **16.1** | <1 | <1 |
| 105 | = | 62 | BM |  | 51.2 |  | <1 | <1 | <1 | n.a. | **47.8** | **15.4** | <1 | 1.3 | 6 |
| = | = | = | THY |  |  | 81 | **78.2** | <1 | 21.3 | <1 | n.a. | n.a. | n.a. | n.a. | n.a. |
| = | = | = | SPL | 340 | 35 |  | 9 | 2 | <1 | n.a. | **24** | **12** | **8** | 20 | 10 |
| = | = | = | LIV | 5.3 | 18.5 |  | 2.9 | 3.2 | <1 | n.a. | **38** | **13.5** | **9.8** | 2 | 1.4 |

|  | | | | | | | % expression of the indicated markers in YFP+GFP- and YFP+GFP+ cell populations as indicated | | | | | | | | |
| --- | --- | --- | --- | --- | --- | --- | --- | --- | --- | --- | --- | --- | --- | --- | --- |
| Id | Type | Time of analysis (days) | Organ | Spleen weight/ Liver inf* | YFP+ GFP- (%) | YFP+ GFP+ (%) | CD4+ CD8- (%) | CD8+ CD4- (%) | CD4+ CD8+ (%) | CD4- CD8- (%) | Gr-1+ CD11b+ (%) | Gr-1- CD11b+ (%) | Gr-1+ CD11b-(%) | CD19+ IgM+ (%) | CD19+ IgM- (%) |
| 106 | FLIP/Myc | = | BM |  | 59.6 |  | <1 | <1 | <1 | n.a. | 35.8 | 9.1 | 1.8 | <1 | <1 |
| = | = | = | THY |  | 85.3 |  | **69.4** | <1 | 28.4 | 1.4 | n.a. | n.a. | n.a. | n.a. | n.a. |
| = | = | = | SPL | 1236 | 34.1 |  | 1.6 | <1 | <1 | n.a. | **79.1** | **14.9** | <1 | 14 | 3 |
| = | = | = | = |  |  | 4.7 | **79.2** | 1.2 | <1 | n.a. | 1.6 | 3.8 | <1 | 2.1 | 1.8 |
| = | = | = | LIV | 8.8 | 34.2 |  | 1 | <1 | <1 | n.a. | **59** | **11.2** | **7.8** | <1 | <1 |
| = | = | = | = |  |  | 5 | **75** | 3.9 | 1.3 | n.a. | *4.8* | *8.4* | <1 | 3.2 | 1.4 |
| 107 | = | 66 | BM |  |  | 24.6 | 1.5 | 1.5 | <1 | n.a. | **63.6** | 6.8 | 1.5 | 1.7 | 5.8 |
| = | = | = | THY |  |  | 81.4 | 10.3 | 4.8 | 83.2 | 1.7 | n.a. | n.a. | n.a. | n.a. | n.a. |
| = | = | = | SPL | 352 |  | 16 | **45** | 2 | 1 | n.a. | *16* | 2 | **28** | 5 | <1 |
| = | = | = | LIV | 7.8 |  | 13.4 | **15.8** | *9.8* | <1 | n.a. | **23** | **15.8** | **8** | 8.4 | <1 |

**Table S1d: Flow cytometric analysis of bone marrow, thymus, spleen and liver in Bcl-x_L_-GFP/Myc-YFP recipient mice (DBA/2).**

|  | | | | | | | % expression of the indicated markers in YFP+GFP- and YFP+GFP+ cell populations as indicated | | | | | | | | |
| --- | --- | --- | --- | --- | --- | --- | --- | --- | --- | --- | --- | --- | --- | --- | --- |
| Id | Type | Time of analysis (days) | Organ | Spleen weight/ Liver inf* | YFP+ GFP- (%) | YFP+ GFP+ (%) | CD4+ CD8- (%) | CD8+ CD4- (%) | CD4+ CD8+ (%) | CD4- CD8- (%) | Gr-1+ CD11b+ (%) | Gr-1- CD11b+ (%) | Gr-1+ CD11b-(%) | CD19+ IgM+ (%) | CD19+ IgM- (%) |
| 128 | Bclx_L_/Myc | 16 | BM |  |  | 85.7 | <1 | <1 | <1 | n.a. | **71.2** | 7.3 | <1 | <1 | <1 |
| = | = | = | THY |  |  | 0.5 | n.a. | n.a. | n.a. | n.a. | 1.8 | 2.6 | 3 | n.a. | n.a. |
| = | = | = | SPL | 414 |  | 54.9 | <1 | <1 | <1 | n.a. | **60.6** | 5.5 | <1 | 1.1 | 2.6 |
| = | = | = | LIV | 14.8 |  | 69.1 | <1 | <1 | <1 | n.a. | **68.9** | *7* | <1 | <1 | <1 |
| 131 | = | = | BM |  |  | 89.3 | <1 | <1 | <1 | n.a. | **58.1** | *11.8* | <1 | 5.5 | <1 |
| = | = | = | THY |  |  | 0.7 | n.a. | n.a. | n.a. | n.a. | n.a. | n.a. | n.a. | n.a. | n.a. |
| = | = | = | SPL | 480 |  | 43.1 | <1 | 1.2 | <1 | n.a. | **48.2** | 8.8 | <1 | 2.5 | 3.2 |
| = | = | = | LIV | 14.9 |  | 72.6 | 1 | <1 | <1 | n.a. | **46.9** | **13** | <1 | <1 | 1.1 |
| 132 | = | = | BM |  |  | 89.4 | <1 | 1.2 | <1 | n.a. | **58.4** | *11.6* | <1 | 1.2 | 11.6 |
| = | = | = | THY |  |  | 9.9 | 1.8 | <1 | 91.3 | 3.4 | 1.1 | 1.7 | <1 | n.a. | n.a. |
| = | = | = | SPL | 428 |  | 57.2 | <1 | 1.1 | <1 | n.a. | **60.7** | 9.4 | <1 | 2.4 | 2.6 |
| = | = | = | LIV | 15.1 |  | 77.1 | 1 | <1 | <1 | n.a. | **54.9** | **11.9** | <1 | <1 | <1 |
| 133 | = | 17 | BM |  |  | 92.5 | 1.3 | <1 | <1 | n.a. | *45.3* | **34.3** | <1 | <1 | 7.6 |
| = | = | = | THY |  |  | 6.2 | 1.2 | 92.9 | 91.3 | 3.2 | <1 | <1 | 2.1 | n.a. | n.a. |
| = | = | = | SPL | 434 |  | 52.5 | <1 | 1.4 | <1 | n.a. | **37.2** | **20.6** | <1 | 3.2 | 3 |
| = | = | = | LIV | 20.1 |  | 71.9 | <1 | <1 | <1 | n.a. | **38.8** | **25.4** | <1 | 1.6 | 1.2 |
| 134 | = | = | BM |  |  | 92 | <1 | <1 | <1 | n.a. | *49.2* | **30.2** | <1 | 1.2 | 10.8 |
| = | = | = | THY |  |  | 2.8 | 4.7 | 1.4 | 82.5 | 6.6 | *6.2* | *4.5* | 6.9 | n.a. | n.a. |
| = | = | = | SPL | 336 |  | 49.1 | <1 | 3 | <1 | n.a. | **49.5** | **12.1** | <1 | 3.2 | 3.1 |
| = | = | = | LIV | 22.3 |  | 82 | <1 | <1 | <1 | n.a. | **41.4** | **20.9** | <1 | 1.1 | <1 |

|  | | | | | | | % expression of the indicated markers in YFP+GFP- and YFP+GFP+ cell populations as indicated | | | | | | | | |
| --- | --- | --- | --- | --- | --- | --- | --- | --- | --- | --- | --- | --- | --- | --- | --- |
| Id | Type | Time of analysis (days) | Organ | Spleen weight/ Liver inf* | YFP+ GFP- (%) | YFP+ GFP+ (%) | CD4+ CD8- (%) | CD8+ CD4- (%) | CD4+ CD8+ (%) | CD4- CD8- (%) | Gr-1+ CD11b+ (%) | Gr-1- CD11b+ (%) | Gr-1+ CD11b-(%) | CD19+ IgM+ (%) | CD19+ IgM- (%) |
| 135 | Bclx_L_/Myc | 17 | BM |  |  | 91.5 | <1 | <1 | <1 | n.a. | **49.2** | **30.2** | <1 | 1.2 | 10.8 |
| = | = | = | THY |  |  | 2.8 | 4.7 | 1.4 | 82.5 | 6.6 | *6.2* | *4.5* | *6.9* | n.a. | n.a. |
| = | = | = | SPL | 510 |  | 49.8 | 2.1 | 1.8 | <1 | n.a. | **38.4** | **14.8** | <1 | 3.2 | 3 |
| = | = | = | LIV | 22.3 |  | 82 | <1 | <1 | <1 | n.a. | **41.4** | **20.9** | <1 | 1.1 | <1 |
| 138 | = | 18 | BM |  |  | 91 | <1 | <1 | <1 | n.a. | **69.4** | *13.1* | <1 | <1 | 6.6 |
| = | = | = | THY |  |  | 0.8 | <1 | <1 | 93 | 2.1 | 1.3 | 4.1 | *22* | n.a. | n.a. |
| = | = | = | SPL | 572 |  | 50.9 | <1 | 2.2 | <1 | n.a. | **61.2** | **10.4** | <1 | 4.2 | 3.6 |
| = | = | = | LIV | 9 |  | 59.8 | n.a. | n.a. | n.a. | n.a. | **45.1** | **17.3** | <1 | n.a. | n.a. |
| 139 | = | = | BM |  |  | 86.7 | n.a. | n.a. | n.a. | n.a. | **62.6** | **20.8** | <1 | <1 | 5.6 |
| = | = | = | THY |  |  | 0.3 | n.a. | n.a. | n.a. | n.a. | 1.5 | 2.8 | *33.1* | n.a. | n.a. |
| = | = | = | SPL | 440 |  | 43.1 | <1 | 3.1 | <1 | n.a. | **57.2** | **12.6** | <1 | 3.8 | 1.8 |
| = | = | = | LIV | 8.8 |  | 56.6 | n.a. | n.a. | n.a. | n.a. | **49.6** | **12.9** | <1 | n.a. | n.a. |
| 140 | = | = | BM |  |  | 89.9 | n.a. | n.a. | n.a. | n.a. | **55.9** | **12.4** | <1 | 2 | 15.2 |
| = | = | = | THY |  |  | 17.8 | <1 | <1 | 95.3 | 2.9 | <1 | <1 | <1 | n.a. | n.a. |
| = | = | = | SPL | 594 |  | 55.9 | <1 | <1 | <1 | n.a. | **65.4** | 9.6 | <1 | 2 | 1.9 |
| = | = | = | LIV | 12.8 |  | 71.9 | n.a. | n.a. | n.a. | n.a. | **60.7** | **14.2** | 1.3 | n.a. | n.a. |
| 141 | = | = | BM |  |  | 89.3 | n.a. | n.a. | n.a. | n.a. | **65.6** | **21.2** | <1 | <1 | 3.8 |
| = | = | = | THY |  |  | 2 | <1 | <1 | 91.8 | 4.8 | 1 | 2.1 | 8.1 | n.a. | n.a. |
| = | = | = | SPL | 631 |  | 49.4 | <1 | 2.5 | <1 | n.a. | **56.8** | **13.2** | <1 | 2.9 | 2.4 |
| = | = | = | LIV | 22.3 |  | n.a. | n.a. | n.a. | n.a. | n.a. | n.a. | n.a. | n.a. | n.a. | n.a. |
| 142 | = | 18 | BM |  |  | 91.2 | n.a. | n.a. | n.a. | n.a. | **60.3** | **21.9** | <1 | 1.1 | 3.9 |
| = | = | = | THY |  |  | 0.8 | n.a. | n.a. | n.a. | n.a. | 6.2 | 4.5 | 6.9 | n.a. | n.a. |
| = | = | = | SPL | 531 |  | 46.4 | 1 | 2 | <1 | n.a. | **56.6** | **11.3** | <1 | 3.5 | 2.1 |
| = | = | = | LIV | 13.6 |  | 66.9 | n.a. | n.a. | n.a. | n.a. | **54.5** | **19.3** | <1 | n.a. | n.a. |

**Table S1e: Flow cytometric analysis of bone marrow, thymus, spleen and liver in Bcl-x_L_-GFP/Myc-YFP recipient mice. (BALB/c)**

|  | | | | | | | % expression of the indicated markers in YFP+GFP- and YFP+GFP+ cell populations as indicated | | | | | | | | |
| --- | --- | --- | --- | --- | --- | --- | --- | --- | --- | --- | --- | --- | --- | --- | --- |
| Id | Type | Time of analysis (days) | Organ | Spleen weight/ Liver inf* | YFP+ GFP- (%) | YFP+ GFP+ (%) | CD4+ CD8- (%) | CD8+ CD4- (%) | CD4+ CD8+ (%) | CD4- CD8- (%) | Gr-1+ CD11b+ (%) | Gr-1- CD11b+ (%) | Gr-1+ CD11b-(%) | CD19+ IgM+ (%) | CD19+ IgM- (%) |
| 148 | Bclx_L_/Myc | 14 | BM |  |  | 79.9 | <1 | <1 | 2.4 | n.a. | n.a. | n.a. | n.a. | 11.3 | <1 |
| = | = | = | THY |  |  | 17.5 | 1.6 | <1 | 85 | 5.6 | <1 | <1 | 2.1 | n.a. | n.a. |
| = | = | = | SPL | 748 |  | 54.1 | 2.8 | 1.3 | <1 | n.a. | **68.3** | **10.6** | <1 | <1 | 1.6 |
| = | = | = | LIV | 27.4 |  | 79.1 | <1 | <1 | <1 | n.a. | **41.9** | **18.6** | <1 | <1 | <1 |
| 149 | = | = | BM |  |  | 80.3 | <1 | <1 | <1 | n.a. | **56.2** | *10.5* | 1.5 | <1 | 6.9 |
| = | = | = | THY |  |  | 12.7 | 0.7 | 2.5 | 59.7 | 29.7 | *13* | *9* | *9.8* | n.a. | n.a. |
| = | = | = | SPL | 640 |  | 47.5 | <1 | 1 | <1 | n.a. | **62.3** | 6.4 | <1 | <1 | <1 |
| = | = | = | LIV | 40.5 |  | 81.2 | <1 | <1 | <1 | n.a. | **30.2** | **21.5** | <1 | <1 | <1 |
| 150 | = | = | BM |  |  | 82.5 | <1 | <1 | <1 | n.a. | **66.7** | 8.8 | 1.4 | <1 | 5.4 |
| = | = | = | THY |  |  | 2 | <1 | 3.3 | 69.5 | 17.3 | *8* | *5.8* | *26.7* | n.a. | n.a. |
| = | = | = | SPL | 726 |  | 58 | <1 | 1.1 | <1 | n.a. | **66.9** | 5.7 | <1 | <1 | 1.2 |
| = | = | = | LIV | 33.8 |  | 79 | <1 | <1 | <1 | n.a. | **44.5** | **15.7** | <1 | <1 | <1 |
| 151 | = | 15 | BM |  |  | 81.8 | <1 | <1 | <1 | n.a. | **60.4** | 9.4 | 2.1 | <1 | 10 |
| = | = | = | THY |  |  | 10.9 | 2.8 | 3.3 | 74.7 | 10.5 | *12.4* | *5.2* | *16.1* | n.a. | n.a. |
| = | = | = | SPL | 728 |  | 52.2 | <1 | 1.1 | <1 | n.a. | **72.3** | 6.7 | <1 | <1 | 1.2 |
| = | = | = | LIV | 25.7 |  | 73.1 | <1 | <1 | <1 | n.a. | **55.2** | **11.2** | 1.1 | <1 | <1 |
| 152 | = | = | BM |  |  | 82.3 | <1 | <1 | <1 | n.a. | **59.5** | 7.1 | 1.8 | 1.2 | 10.2 |
| = | = | = | THY |  |  | 12.6 | 3.3 | 1.7 | 51.7 | 29.2 | *13.4* | *4* | *11.6* | n.a. | n.a. |
| = | = | = | SPL | 698 |  | 50 | <1 | 1.2 | <1 | n.a. | **71.2** | 6.8 | <1 | <1 | 1.2 |
| = | = | = | LIV | 28.7 |  | 74.8 | 1.6 | <1 | <1 | n.a. | **51.2** | **12.3** | 1.1 | <1 | <1 |

|  | | | | | | | % expression of the indicated markers in YFP+GFP- and YFP+GFP+ cell populations as indicated | | | | | | | | |
| --- | --- | --- | --- | --- | --- | --- | --- | --- | --- | --- | --- | --- | --- | --- | --- |
| Id | Type | Time of analysis (days) | Organ | Spleen weight/ Liver inf* | YFP+ GFP- (%) | YFP+GFP+ (%) | CD4+ CD8- (%) | CD8+ CD4- (%) | CD4+ CD8+ (%) | CD4- CD8- (%) | Gr-1+ CD11b+ (%) | Gr-1- CD11b+ (%) | Gr-1+ CD11b-(%) | CD19+ IgM+ (%) | CD19+ IgM- (%) |
| 153 | Bclx_L_/Myc | = | BM |  |  | 86.2 | 1.1 | <1 | <1 | n.a. | **60.4** | 7.2 | 2 | <1 | 9.4 |
| = | = | = | THY |  |  | 4 | 1.7 | 1.7 | 63.5 | 18.8 | *5* | *9.4* | *22.8* | n.a. | n.a. |
| = | = | = | SPL | 722 |  | 49.3 | <1 | 1.2 | <1 | n.a. | **61.3** | 7.7 | <1 | <1 | 2.3 |
| = | = | = | LIV | 26.6 |  | 74.2 | <1 | <1 | <1 | n.a. | **40.9** | **13.8** | 1.2 | <1 | 1.1 |
| 154 | = | 15 | BM |  |  | 88.7 | <1 | <1 | <1 | n.a. | **70.1** | 8.3 | 1.7 | <1 | 4 |
| = | = | = | THY |  |  | 7.7 | 2 | 2.5 | 57.3 | 23.7 | *9.6* | *7.6* | *9* | n.a. | n.a. |
| = | = | = | SPL | 868 |  | 49.9 | <1 | <1 | <1 | n.a. | **67.8** | 7.9 | <1 | <1 | 1.3 |
| = | = | = | LIV | 41.3 |  | 81.6 | <1 | <1 | <1 | n.a. | **43** | **13.7** | 1 | <1 | <1 |
| 156 | = | 18 | BM |  |  | 81.1 | <1 | <1 | <1 | n.a. | 27.3 | 9.5 | 2.3 | 1.5 | 26.7 |
| = | = | = | THY |  |  | 18.5 | 4.2 | 5.1 | 55 | 23.8 | *9.1* | *5.4* | *18.9* | n.a. | n.a. |
| = | = | = | SPL | 864 |  | 48.9 | <1 | <1 | <1 | n.a. | **55.8** | 6.9 | <1 | 1.3 | 5.9 |
| = | = | = | LIV | 29.1 |  | 62.5 | <1 | <1 | <1 | n.a. | **34.4** | **13.5** | 1.7 | <1 | 2.7 |
| 157 | = | = | BM |  |  | 91.6 | 1.8 | <1 | 3.7 | n.a. | 21.4 | **16.4** | 2.5 | <1 | 15.9 |
| = | = | = | THY |  |  | 13.3 | 4.4 | 2.2 | 47.2 | 35.2 | *4.7* | *7.3* | *17.1* | n.a. | n.a. |
| = | = | = | SPL | 820 |  | 46.8 | <1 | <1 | <1 | n.a. | **58.7** | **9.9** | <1 | <1 | 2 |
| = | = | = | LIV | 36.9 |  | 69.2 | <1 | <1 | <1 | n.a. | **39** | **18.7** | 1.2 | <1 | 1.5 |

**Table S1f: Flow cytometric analysis of bone marrow, thymus, spleen and liver in Bcl-2-GFP/Myc-YFP recipient mice. (BALB/c)**

|  | | | | | | | % expression of the indicated markers in YFP+GFP- and YFP+GFP+ cell populations as indicated | | | | | | | | |
| --- | --- | --- | --- | --- | --- | --- | --- | --- | --- | --- | --- | --- | --- | --- | --- |
| Id | Type | Time of analysis (days) | Organ | Spleen weight/ Liver inf* | YFP+ GFP- (%) | YFP+ GFP+ (%) | CD4+ CD8- (%) | CD8+ CD4- (%) | CD4+ CD8+ (%) | CD4- CD8- (%) | Gr-1+ CD11b+ (%) | Gr-1- CD11b+ (%) | Gr-1+ CD11b-(%) | CD19 IgM+ (%) | CD19+ IgM- (%) |
| 191 | Bcl-2/Myc | 17 | BM |  |  | 60.6 | <1 | <1 | <1 | n.a. | *32.4* | *14* | *4.1* | <1 | 10.2 |
| = | = | = | THY |  |  | 0.4 | n.a. | n.a. | n.a. | n.a. | n.a. | n.a. | n.a. | n.a. | n.a. |
| = | = | = | SPL | 1140 |  | 41.8 | <1 | 2 | <1 | n.a. | **45** | **12.1** | 4.8 | 1.9 | 2.1 |
| = | = | = | LIV | 15.9 |  | 60.6 | <1 | <1 | <1 | n.a. | **23.7** | **20.6** | 1.6 | 1 | <1 |
| 192 | = | = | BM |  |  | 76.3 | <1 | <1 | <1 | n.a. | **40.5** | **28** | 2.9 | <1 | 2.9 |
| = | = | = | THY |  |  | 7.6 | <1 | <1 | 92.4 | 1.6 | 1.3 | <1 | 1.1 | n.a. | n.a. |
| = | = | = | SPL | 888 |  | 38.1 | <1 | 1.4 | <1 | n.a. | **42.2** | **13.3** | 3.6 | 1.5 | 1 |
| = | = | = | LIV | 20.6 |  | 65.5 | <1 | <1 | <1 | n.a. | **23.9** | **23.9** | 1.7 | <1 | <1 |
| 193 | = | = | BM |  |  | 65.3 | <1 | <1 | <1 | n.a. | **50.3** | **19.5** | 1.6 | 1 | 7.7 |
| = | = | = | THY |  |  | 4.8 | 5.3 | <1 | 82.3 | 3.4 | 2.1 | <1 | 2.2 | n.a. | n.a. |
| = | = | = | SPL | 790 |  | 34.2 | <1 | 1.8 | <1 | n.a. | **44.6** | **14.4** | 3.9 | 2.2 | 1.8 |
| = | = | = | LIV | 20.1 |  | 60.1 | <1 | <1 | <1 | n.a. | **22.5** | **23.3** | 1.7 | 1.8 | <1 |
| 194 | = | 19 | BM |  |  | 50.6 | <1 | <1 | <1 | n.a. | **23.1** | **20.9** | 1.7 | <1 | 3.8 |
| = | = | = | THY |  |  | 1 | n.a. | n.a. | n.a. | n.a. | n.a. | n.a. | n.a. | n.a. | n.a. |
| = | = | = | SPL | 1074 |  | 29.7 | <1 | <1 | <1 | n.a. | **31** | **29.2** | 2.2 | 1.1 | <1 |
| = | = | = | LIV | 18.3 |  | 59.7 | <1 | <1 | <1 | n.a. | **29.6** | **30.1** | 2.3 | <1 | <1 |
| 195 | = | = | BM |  |  | 56 | <1 | <1 | <1 | n.a. | **34.1** | **16.7** | 4.5 | <1 | 2.7 |
| = | = | = | THY |  |  | 4.8 | <1 | 1.9 | 72 | 13 | n.a. | n.a. | n.a. | n.a. | n.a. |
| = | = | = | SPL | 894 |  | 28.8 | <1 | <1 | <1 | n.a. | **31.3** | **29.2** | 1.8 | 1.3 | <1 |
| = | = | = | LIV | 22 |  | 61.3 | <1 | <1 | <1 | n.a. | **28.3** | **28.9** | 1.6 | <1 | <1 |

|  | | | | | | | % expression of the indicated markers in YFP+GFP- and YFP+GFP+ cell populations as indicated | | | | | | | | |
| --- | --- | --- | --- | --- | --- | --- | --- | --- | --- | --- | --- | --- | --- | --- | --- |
| Id | Type | Time of analysis (days) | Organ | Spleen weight/ Liver inf* | YFP+ GFP- (%) | YFP+ GFP+ (%) | CD4+ CD8- (%) | CD8+ CD4- (%) | CD4+ CD8+ (%) | CD4- CD8- (%) | Gr-1+ CD11b+ (%) | Gr-1- CD11b+ (%) | Gr-1+ CD11b-(%) | CD19+ IgM+ (%) | CD19+ IgM- (%) |
| 196 | Bcl-2/Myc | 19 | BM |  |  | 53.4 | <1 | <1 | <1 | n.a. | *34.3* | **34.4** | 3.3 | <1 | 1.3 |
| = | = | = | THY |  |  | 0.7 | n.a. | n.a. | n.a. | n.a. | n.a. | n.a. | n.a. | n.a. | n.a. |
| = | = | = | SPL | 970 |  | 31.8 | <1 | <1 | <1 | n.a. | **19.1** | **38.1** | <1 | <1 | <1 |
| = | = | = | LIV | 22.1 |  | 63.9 | <1 | <1 | <1 | n.a. | **23.6** | **29.1** | 2.9 | <1 | <1 |
| 197 | = | = | BM |  |  | 57.5 | <1 | <1 | <1 | n.a. | 28.5 | **13.6** | 2.8 | <1 | 19.5 |
| = | = | = | THY |  |  | <1 | n.a. | n.a. | n.a. | n.a. | n.a. | n.a. | n.a. | n.a. | n.a. |
| = | = | = | SPL | 940 |  | 32.6 | <1 | 1.2 | <1 | n.a. | **22.5** | **41.2** | <1 | 1 | 2.2 |
| = | = | = | LIV | 25.4 |  | 63.5 | <1 | <1 | <1 | n.a. | **22.8** | **23.9** | 2.9 | <1 | <1 |

# % YFP^+^GFP^-^, YFP^+^GFP^+^ and YFP^-^GFP^-^ populations have been pooled and analyzed for expression of the indicated markers. * organ weight in mg. n.a = not analyzed. Numbers in bold indicate severely abnormal values (Numbers in bold have been used to construct summary Fig. 8). Numbers in italic indicate abnormal values.
