# Supplementary material for: Inhibition of the Intrinsic but Not the Extrinsic Apoptosis Pathway Accelerates and Drives Myc-Driven Tumorigenesis Towards Acute Myeloid Leukemia
Source: PLoS One. 2012 Feb 29;7(2):e31366. doi: 10.1371/journal.pone.0031366 (PMC3290626; doi:10.1371/journal.pone.0031366)
Supplement: Table S2 — Differential count of nucleated cells in peripheral blood smears in MYC/BCL-XL and control mice. (DOCX) [file pone.0031366.s011.docx]

**Table S2. Differential count of nucleated cells in peripheral blood smears in MYC/BCL-XL and control mice.**

| **Animals transplanted**  **with HSC expressing*** | **Percentages of nucleated cells in blood** | | | | | | |
| --- | --- | --- | --- | --- | --- | --- | --- |
|  | blasts | promyelocytes | myelocytes | bands | polymorphs | monocytes | mature |
|  |  |  |  |  |  |  | lymphocytes |
|  |  |  |  |  |  |  |  |
| Mock/Mock #1 | 0 | 0 | 0 | 6,5 | 31,5 | 5 | 57 |
| Mock/Mock #2 | 1,5 | 0 | 0 | 7 | 14 | 2,5 | 75 |
| Mock/Mock #3 | 0,5 | 0 | 0 | 6,5 | 33,5 | 3 | 56,5 |
| MYC/BCL-X_L_ #1 | 58 | 3,5 | 1,5 | 16 | 9,5 | 1,5 | 10 |
| MYC/BCL-X_L_ #2 | 57,5 | 6,5 | 6,5 | 10 | 6,5 | 3,5 | 6,5 |
| MYC/BCL-X_L_ #3 | 48,5 | 8,5 | 8 | 19 | 9 | 2 | 5 |

* Results represent counts from individual mice. Numbers correspond to the identity of the individual animal.
